# Neurological complications post aortic arch surgery: a state of art review

**DOI:** 10.1186/s13019-025-03706-1

**Published:** 2025-12-17

**Authors:** Toufik Abdul-Rahman, Jann Ludwig Mueller-Gomez, Aditya Gaur, Miranda Llama Luna, Marcos Lisbona-Buzali, Ranferi Eduardo Herrera-Calderón, Hala Ibrahim Thaalibi, Poulami Roy, Tamara Mena-Guerrero, Mirza Ammar Arshad, Manahil Mustajab, Mrinmoy Kundu, Sulagna Nag Chowdhury, Andrew Awuah Wireko, Oday Atallah

**Affiliations:** 1 Department of Research, Toufik’s World Medical Association, Antonova 10, Sumy, 40007 Ukraine; 2 Center for Research in Health Sciences (CICSA), Faculty of Medicine, Anahuac University North Campus, Huixquilucan, Mexico; 3 Yeovil District Hospital, Somerset NHS Foundation Trust, Higher Kingston, Yeovil, UK; 4 Beirut Arab University Faculty of Medicine, Beirut, Lebanon; 5 Department of Medicine, North Bengal Medical College and Hospital, Siliguri, India; 6 Rahbar Medical and Dental College, Lahore, Pakistan; 7 Dow Medical College, Karachi, Pakistan; 8 Institute of Medical Sciences, SUM Hospital, Bhubaneswar, India; 9 Cardiothoracic and Vascular Surgery department, Kolkata, West Bengal India; 10 Department of Neurosurgery, Carl Von Ossietzky University Oldenburg, Oldenburg, Germany

**Keywords:** Neurological complications, Stroke, Neurocognitive decline, Aortic arch surgery, Hemiarch surgery, Total arch replacement

## Abstract

Aortic arch surgery is a complex and high-risk operation undertaken to correct aneurysms, dissections, and traumatic aortic injuries. Despite notable improvement in surgical technique, perioperative care, and neuroprotection, the risk of neurological complications remains a predominant cause of concern. Such complications, which include permanent and transient neurological deficits, spinal cord damage, intellectual dysfunction, and seizures, are caused by conditions like cerebral hypoperfusion, embolism, reperfusion injury, and systemic inflammatory responses. The review seeks to summarize available evidence to cover the incidence, risk factors, mechanisms, prevention, and management of neurological complications in aortic arch surgery. It also evaluates the effectiveness of preventative strategies such as selective antegrade cerebral perfusion, hypothermia, intraoperative monitoring, and new pharmacologic approaches (i.e., hypertonic saline dextran, thiopental) in reducing neurological risk. Despite advances, there are important gaps in the management of long-term complications, reflecting the need for ongoing innovation in surgical and perioperative care. This review is a summary to assist clinicians in decreasing adverse outcomes in this high-risk group of patients.

## Introduction

The aortic arch is one of the five main segments of the aorta and extends from the innominate artery to the left subclavian artery. One of its functions is to give off branches that supply the brain and upper limbs [1, 2]. Aortic arch disease, often involving aneurysms and dissections, typically requires partial or complete arch replacement to prevent life-threatening complications [3]. These surgeries are highly complex and involve periods of circulatory arrest, which place significant stress on the body [4, 5]. Despite advances in surgical techniques and perioperative care—including anesthesia, perfusion, and intensive care—aortic arch surgeries still pose significant risks of morbidity and mortality [6–9]. In emergency cases, the mortality rate can be as high as 50%, while for elective procedures, the rate is lower but still significant at approximately 9% [10, 11]. Additionally, about 25% of patients may need reintervention due to severe postoperative complications [6].

Thoracic aortic aneurysms, the second most common thoracic aortic condition, are often asymptomatic until rupture or dissection occurs [12]. Surgical decisions must carefully balance the risks of intervention with the progression of the disease. These risks depend on patient-specific factors like comorbidities and anatomy, as well as disease-related factors such as family history and aneurysm type [10]. Current guidelines from both American and European medical societies recommend surgery when the aortic arch diameter reaches 5.5 cm [13, 14]. Other factors, including symptoms, growth rate, patient size, and the presence of pseudoaneurysms, also play a role in determining the timing of surgery [10].

Aortic dissection is a life-threatening cardiovascular emergency that requires immediate attention. It is classified as either Stanford type A or type B, depending on where the dissection occurs. Stanford type A involves the ascending aorta and often extends into the arch, typically requiring urgent surgical intervention [15–17]. Without treatment, the mortality rate for Stanford type A dissections can reach up to 50% within the first 48 h [10].

Aortic arch surgery carries a significant risk of complications, with neurological injury being one of the most critical. Brain injuries following these procedures can be categorized as either permanent or temporary [3, 18, 19]. Given the variability in definitions across the literature, this study adopts the definitions used in accordance with established surgical databases and contemporary literature. Permanent neurologic dysfunction (PND) is defined as a new and permanent focal neurologic deficit with or without evidence of cerebral infarction on computed tomography or magnetic resonance imaging and confirmed by a neurologist [1]. This includes focal issues, such as embolic stroke, or global deficits like coma, which persist at discharge and are detectable on CT imaging [3, 19–21]. Temporary neurologic dysfunction (TND) is defined as postoperative confusion, delirium, obtundation, or transient focal deficits with resolution within 24 h and negative brain computed tomography or magnetic resonance imaging scans [1]. Confusion or delirium typically resolves before discharge and shows no abnormalities on imaging [3, 19, 20, 22, 23]. Despite advancements in cerebral protection strategies, postoperative neurological deficits remain common. Both TND and PND, although caused by different mechanisms, significantly impact patient outcomes, often leading to prolonged hospital stays and, in severe cases, increased mortality [18, 19, 24–27]. The incidence of PND ranges from 0.8% to 28%, while TND occurs in 3.3% to 28% of cases [25, 27–34].

Several risk factors for postoperative neurological deficits have been identified, including preoperative neurological symptoms, disturbances in consciousness, and hemodynamic instability [19, 35]. Other factors that increase the risk of brain injury include advanced age, a history of stroke, carotid plaque or stenosis, emergency surgery, renal dysfunction, hypotension, prolonged aortic clamping time, the duration of deep hypothermic circulatory arrest (DHCA), postoperative hypoxemia, low cardiac output syndrome, and large-volume blood transfusions [17, 23, 36–39].

Neurological injury during the aortic arch and ascending aorta surgery can occur through several mechanisms. Emboli may be dislodged during dissection or vessel manipulation, while global hypoperfusion can result from prolonged ischemia due to circulatory arrest, cerebral vasospasm lasting up to eight hours post-HCA, or unstable hemodynamics following cardiopulmonary bypass (CPB) [3, 23, 40]. Other contributing factors include edema from venous congestion, ischemia-reperfusion injury, or even the protective strategies themselves [23]. Infarction typically develops over 72 h, and perioperative care—particularly in ventilation, fluid balance, glucose control, and temperature management—is crucial to patient outcomes [41].

Given these risks, protective strategies are essential for managing patients undergoing aortic arch surgery. Intraoperative monitoring plays a key role in preventing neurological impairment, although no reliable neuroprotective agents are currently available, and brain monitoring techniques have a limited ability to detect early issues. Effective brain protection relies on three main factors: the duration of circulatory arrest, brain temperature during arrest, and cerebral perfusion [3].

Despite advancements in surgical techniques and intraoperative monitoring, neurological complications after aortic arch surgery remain a significant challenge, with a substantial impact on long-term morbidity and mortality. As a result, there has been an increasing focus on postoperative care, particularly neuroprotective strategies, as a key approach to reducing these complications [42]. This article aims to provide a comprehensive, up-to-date review of the neurological issues related to aortic arch surgery, including their incidence, risk factors, pathophysiology, prevention, and management with an emphasis on perioperative management. It will also explore current practices and potential future improvements in patient outcomes.

## Methodology

This narrative review employed a comprehensive search strategy across multiple electronic databases, including PubMed, Scopus, and Google Scholar, covering literature from the inception of each database to the present (Table 1). The search utilized the following key terms: “neurological complications,” “neurocognitive dysfunction,” “neurological injury,” “stroke,” “aortic arch surgery,” “aortic arch replacement,” “thoracic aortic surgery,” “postoperative,” “post surgery,” and “after surgery.” Boolean operators (AND, OR) were used to refine the search and ensure a thorough exploration of both human and animal studies related to neurological complications following aortic arch surgery.

In addition to database searches, the reference lists of included articles and relevant journals were manually screened to identify further studies of interest. The inclusion criteria encompassed a range of study designs, including experimental studies, observational studies, systematic reviews, and meta-analyses. Studies were considered eligible if they investigated neurological complications following aortic arch surgery, including their mechanisms, prevention, or management strategies. Only articles published in English were included. Unpublished studies and those published in languages other than English were excluded.

**Table 1:** Summary table of methodology for this narrative review

Methodology steps	Description
Search terms	• Use of keywords such as “neurological complications,” “neurocognitive dysfunction,” “neurological injury,” “stroke,” “aortic arch surgery,” “aortic arch replacement,” “thoracic aortic surgery,” “postoperative,” “post surgery,” and “after surgery.” • Use of Boolean operators (AND, OR)
Databases searched	• PubMed, Scopus, Google Scholar
Inclusion criteria	• Articles published entirely in English • Emphasis on neurological complications following aortic arch surgery, including their mechanisms, prevention, or management strategies • Human and animal studies • Various study designs, such as experimental studies, observational studies, systematic reviews, and meta-analyses.
Exclusion criteria	• Articles published in languages other than English • Unpublished studies
Additional search	• Thorough inspection of references mentioned in recent reviews focused on specific diseases through manual examination • Manual search of journal websites • No predefined restriction on the number of studies to be considered

## Aortic arch surgery: overview

### Indications for surgery

Aortic arch surgery is indicated for a variety of conditions, including aortic arch aneurysm, intramural hematoma (IMH), type A aortic dissection, aortic coarctation, and traumatic injuries to the aorta. Aortic aneurysms can arise from numerous etiologies, with Marfan syndrome being the most commonly associated condition. However, recent literature has identified the occurrence of aortic aneurysms in individuals without Marfan syndrome, suggesting a potential genetic predisposition, particularly when these aneurysms manifest in a familial pattern affecting first-degree relatives. Aortic aneurysms are known for their potential for rapid growth, typically expanding at a rate of approximately 0.10 cm per year, with an acceleration of growth as the aneurysm increases in size. The critical threshold for rupture, often referred to as the “hinge point,” is approximately 6.0 cm for the ascending aorta and aortic arch, and 7.0 cm for the descending aorta. Rupture has been documented in 31% of patients by the time the aneurysm reaches these dimensions. Given the high mortality rate of 2.5% and 8% risk of stroke associated with ascending aortic and aortic arch aneurysms, as well as the 8% mortality rate with a similar stroke risk for descending aortic aneurysms, a risk-benefit analysis strongly favors early surgical intervention. In asymptomatic patients, elective surgery is recommended when the aneurysm reaches 5.0 cm in the ascending aorta and 6.5 cm in the descending aorta. Conversely, immediate surgical intervention is advised for symptomatic patients [43].

IMH is often considered a precursor to aortic dissection, characterized by the absence of rupture in the tunica intima or formation of a false lumen. IMH typically presents with chest pain and carries a significant risk of progressing to more severe conditions, such as aortic dissection. Aortic dissection occurs when a tear in the intima allows blood to enter the media layer, creating a false lumen that can rapidly extend along the aorta. This can lead to life-threatening complications such as malperfusion syndromes, aortic rupture, and cardiac tamponade. In acute cases, particularly Type A IMH, there is a high likelihood of progression to these life-threatening complications, all of which are associated with increased mortality [44]. The mortality rate for acute IMH is approximately 20%, with a 5-year survival rate of only 43%. Consequently, immediate surgical intervention is recommended for IMH of the ascending aorta (Type A). Additional indications for aortic arch surgery include cases where IMH has progressed to a contained rupture of the ascending aorta, the development of cardiac tamponade, or the presence of a Type A dissection [45].

Coarctation of the aorta is a congenital defect characterized by the narrowing of the aortic lumen, typically located in the proximal thoracic aorta near the ductus arteriosus. Immediate surgical repair of the aortic arch is the gold standard treatment for both coarctation and re-coarctation in children and adults [46]. Despite successful surgical correction, patients remain at significant risk for late cardiovascular complications. These complications include premature coronary artery disease, re-coarctation, left ventricular outflow tract abnormalities, and major aortic wall issues, such as true or false aneurysm formation (particularly at the repair site), rupture, dissection, endarteritis, and fistula development. Aortic wall complications are not uncommon, occurring in approximately 16% of patients, with the incidence of aneurysms at the previous surgical site ranging from 5% to 50%. Many of these complications, especially those involving the aortic wall, necessitate repeat surgery due to the associated high mortality risk [47].

Traumatic thoracic aortic injury is a critical and life-threatening condition frequently seen in trauma medicine. The majority of patients with such injuries die at the scene, and those who reach the hospital often suffer cardiopulmonary arrest shortly after arrival. Due to the high mortality rate associated with untreated thoracic aortic injuries, prompt surgical intervention is essential once the injury is diagnosed, typically through contrast-enhanced computed tomography (CT). Endovascular repair is increasingly preferred over open surgery because of its lower mortality and morbidity rates, especially in the typically young trauma population who often have multiple injuries. Although endovascular repair carries risks, such as false aneurysm formation and aortobronchial or aortoesophageal fistulas, it is considered safer than open repair. Consequently, endovascular treatment has become the preferred approach in managing traumatic thoracic aortic injuries [48].

In recent years, evolving guidelines have further refined criteria for surgical intervention in aortic arch disease. The 2023 EACTS/STS guidelines on aortic arch disease provide updated recommendations based on recent evidence regarding the diagnosis and management of these cases. One of the bigger shifts is the emphasis on using a zone-based system to describe the arch anatomy, which helps tailor decisions more closely to individual patients. The approach being recommended now leans towards personalization—taking into account the person’s anatomy, other health issues, and the risk of surgery in their specific situation [49]. Furthermore, surgical indications according to the guidelines are not solely dependent on the size, but now also incorporate growth rate (>5 mm/year), presence of symptoms, and connective tissue disorders at lower size thresholds (≥ 5.0 cm). For patients with chronic dissection, a one-off repair is suggested once the arch gets beyond 55 mm, or 40 mm if there’s another problem going on, like an aneurysm or abnormalities with blood flow. Another key point from the guideline is the importance of multidisciplinary team involvement. Decision-making should involve cardiac surgeons, vascular surgeons, radiologists, and others to ensure a comprehensive evaluation. Imaging plays a key role in identifying pathology and selecting the most appropriate intervention. In patients who are at higher risk, endovascular or hybrid procedures might be worth considering depending on feasibility [49].

### Surgical techniques

Depending on the nature of the aortic arch pathology and the patient’s overall condition, treatment options may include open surgical repair, endovascular repair, or a hybrid approach. In current practice, endovascular and hybrid techniques have become increasingly common, as they are considered minimally invasive alternatives to traditional open repair—particularly beneficial for patients at high surgical risk or with multiple comorbidities. However, each approach has its own advantages and limitations, and the choice of treatment should be individualized based on anatomical, clinical, and procedural factors.

#### Traditional open repair

**Fig. 1:**
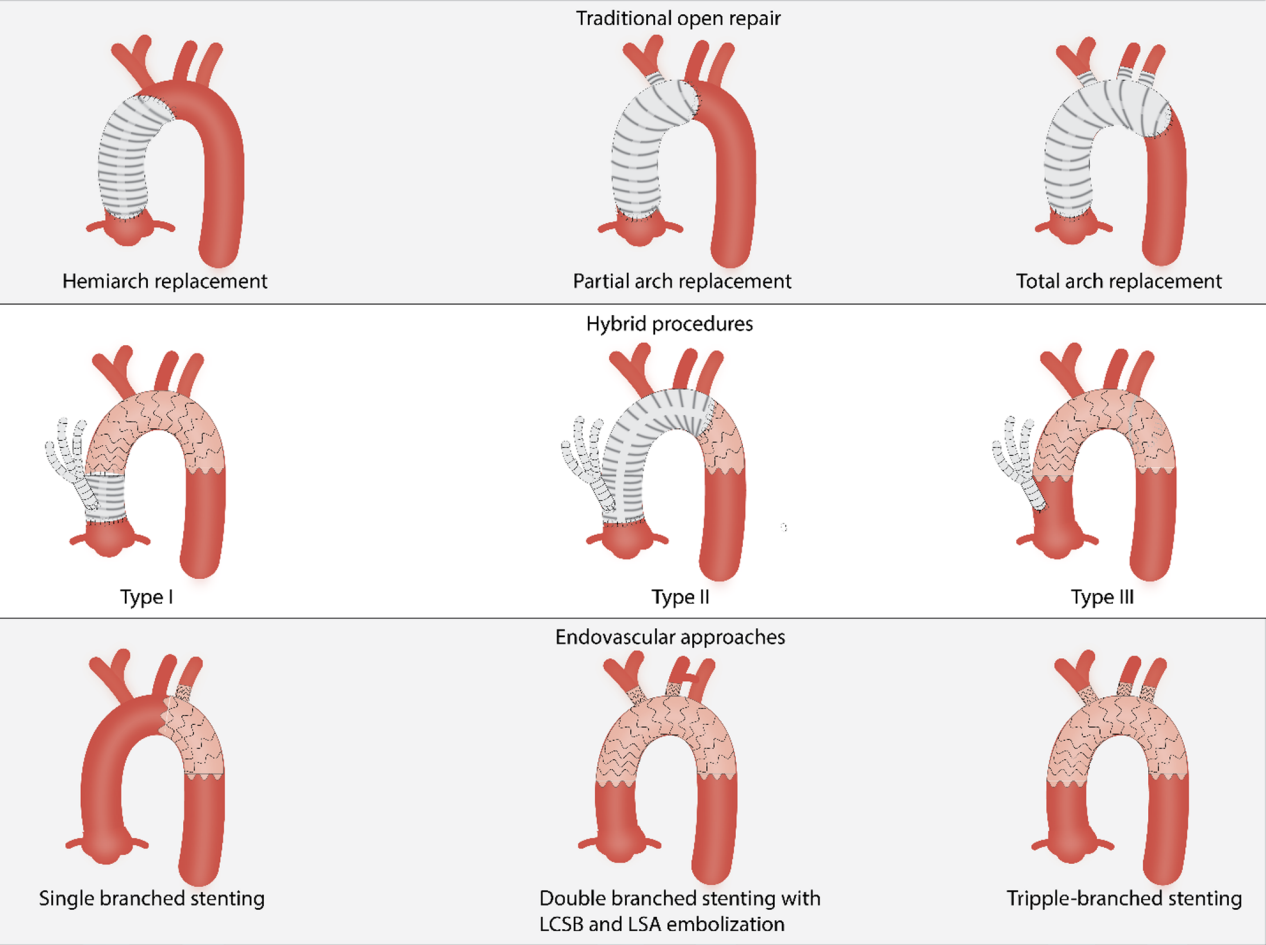
Illustration of Various Surgical Techniques for Aortic Arch Surgery

Open aortic arch replacement is a highly complex procedure that requires the use of CPB, hypothermia, and other adjuncts for neurologic and systemic protection [1]. CPB enables systemic and cerebral perfusion while allowing the patient’s core temperature to be gradually lowered. Hypothermia reduces the metabolic demands of vital organs, providing a protective window during which systemic circulation can be temporarily arrested. During this period, selective cerebral perfusion is often employed to maintain adequate brain oxygenation and minimize the risk of neurological injury. The diseased segment of the aortic arch is then excised and replaced with a synthetic vascular graft. After reconstruction of the arch and reimplantation of the supra-aortic vessels, the patient is gradually rewarmed, systemic perfusion is restored, and cardiac activity is resumed [1, 2]. Open arch replacement can be defined by three types of procedures: Hemiarch, partial arch repair, and total arch replacement (TAR) (Fig. 1).

Hemiarch and partial arch replacement involves replacing the proximal portion of the aortic arch with a surgical graft. However, hemiarch replacement involves only the side of the lesser curvature beyond the level of the innominate artery without the greater curvature, while partial arch replacement involves reconstruction of the innominate artery or both the innominate and left common carotid arteries [50].

TAR is most commonly performed in cases involving the ascending aorta and transverse arch, without extension into the descending thoracic aorta [1, 51]. A typical indication is in patients with previously repaired acute type A dissections that have progressed to form an arch aneurysm. Less commonly, TAR is performed in cases of chronic type B dissection with retrograde extension into the arch, or for isolated aneurysms of the aortic arch, where descending aortic involvement is absent or limited [52]. TAR can be performed through different techniques. Recent ones include the trifurcated technique and the for-branched arch graft technique.

Other surgical approaches have been developed for the treatment of extensive aortic disease involving the distal arch and descending thoracic aorta [51]. Among the most recognized are the elephant trunk (ET) technique, a conventional two-stage procedure, and the frozen elephant trunk (FET) technique, a more recent hybrid approach that combines open and endovascular repair [53]. The ACC/AHA Guidelines recommend ​​the consideration of ET for open surgical repair of an aortic arch aneurysm if the disease extends into the proximal descending thoracic aorta [1]. The ET is indicated in a range of scenarios from elective thoraco-abdominal aneurysm repair to acute aortic dissections, with the aim of curtailing downstream reintervention rates, especially in younger patients and those presenting with aortic arch dilatation or connective tissue diseases [53]. However, ET is associated with graft-related complications, such as graft occlusion and kinking, and neurological complications, including stroke and spinal cord injury (SCI) [53]. To address these limitations, the FET technique was developed as a hybrid approach that combines open and endovascular repair [53]. The FET simplifies the conventional elephant trunk operation, which is primarily a 2-stage procedure, into a 1-stage repair of extended aortic aneurysms [51]. As these two procedures extend to the descending aorta and the focus of this review is limited to aortic arch procedures, the technical details of these approaches will not be discussed in depth.

#### Endovascular approaches

Thoracic endovascular aortic repair (TEVAR), developed in the 1990 s, is a less invasive alternative to open surgical repair for aortic aneurysms [51]. It is particularly desirable for high-risk patients. Compared to open surgery, TEVAR offers the advantages of less surgical trauma, reduced mechanical circulatory support, and avoidance of aortic cross-clamping. These factors contribute to fewer cerebral complications, lower early mortality, and shorter hospital stays [54]. Furthermore, while cerebrovascular accidents remain a concern in open aortic arch repair, studies report an early incidence of only 0 to 5.4% following TEVAR [54, 55]. Despite these advantages, TEVAR in the aortic arch presents unique challenges due to the complex anatomy and limited availability of suitable landing zones for endograft deployment. These limitations can be addressed with advanced devices such as branched or fenestrated grafts, which enable remote debranching of supra-aortic vessels [55]. Another significant technical hurdle is the need to maintain continuous cerebral perfusion during the reconstruction of the innominate and left carotid arteries [54].

Several advanced endovascular strategies have been developed to address the challenges of aortic arch repair, particularly when preserving supra-aortic branch perfusion is necessary. Among these, the chimney technique, fenestrated endografts, and branched endografts offer distinct approaches with specific technical considerations, indications, and limitations. The chimney technique involves the placement of parallel stent grafts into the supra-aortic branches, positioned alongside the main thoracic endoprosthesis to maintain branch perfusion. While this method demands considerable endovascular expertise, it is often more cost-effective than custom-made fenestrated grafts. However, it is associated with higher rates of endoleak due to the gutter between the parallel grafts. Fenestrated endografts are designed with pre-formed openings (fenestrations) that align with the supra-aortic vessels along the greater curvature of the aortic arch, allowing for precise branch preservation while minimizing manipulation and embolic risk. These grafts are typically used when the middle arch (Zone 2) serves as the proximal landing zone. However, they are not suitable for aneurysms that involve the supra-aortic vessels themselves or those located directly on the greater curvature, where alignment is difficult [56, 57]. Branched endografts, another advanced option, are delivered transfemorally through a large-caliber sheath. Branch components are guided into the target vessels—typically the innominate and left common carotid arteries—using traction wires inserted via smaller sheaths introduced through the brachial arteries or directly into the carotid. This technique offers high technical success rates and demonstrates favorable long-term patency outcomes [58].

The left vertebral artery (LVA), which usually originates from the left subclavian artery, is an important artery that requires special consideration in aortic TEVAR. It is particularly relevant in Zone 2 repairs or debranching procedures. LVA coverage without revascularization can increase the risk of posterior circulation stroke, particularly if the right vertebral artery is hypoplastic or occluded [59]. Current EACTS/STS guidelines recommend preoperative imaging of vertebral artery dominance and revascularization (via LVA–left common carotid transposition or bypass) when the LVA is dominant or the right side is compromised [49]. Failure to account for vertebrobasilar perfusion can result in devastating outcomes.

#### Hybrid procedures

A hybrid arch repair procedure combines traditional open arch repair with endovascular techniques [51]. According to the most recent guidelines, patients with an aortic arch aneurysm who are asymptomatic but meet criteria for intervention, but have a high risk from open surgical repair, can be considered for a hybrid or endovascular approach [1].

Due to the need for circulatory arrest and cardiopulmonary bypass, TAR is often contraindicated in high-risk surgical patients. In such cases, hybrid aortic arch repair serves as a less invasive and effective alternative [53]. The hybrid procedure consists basically of debranching the supra-aortic vessels from the arch to the ascending aorta, creating proximal and distal landing zones suitable for the endovascular insertion of the prosthetic graft [55]. The goal is to create extra-anatomic bypasses [51], that allow endovascular exclusion of the diseased aortic arch without the need for full circulatory arrest. There are currently three types of hybrid repair, types I, II, and III, differentiated by how the debranching of the anatomical zones of the aortic arch is approached. Type I involves complete debranching of the supra-aortic vessels to the ascending aorta, creating a landing zone for TEVAR. It can often be performed without CPB or circulatory arrest [55]. Type II is used when the ascending aorta is not suitable as a proximal landing zone. It involves surgical replacement of the ascending aorta (using CPB, but typically without hypothermic circulatory arrest), followed by debranching and stent grafting [51, 55]. Type III is the FET. As previously noted, it is used to treat extensive aortic disease involving the ascending aorta, aortic arch, descending thoracic aorta, and thoracoabdominal segments. It involves total aortic arch replacement combined with the deployment of a stented graft into the proximal descending thoracic aorta, creating a durable landing zone for future interventions. The procedure is performed using CPB with hypothermic circulatory arrest [51, 55, 60]. The FET emerged as a modern, less invasive alternative intervention for selected patients; however, despite its minimally invasive elements, FET remains a technically complex procedure associated with notable perioperative risks and postoperative complications. These include significant rates of acute neurological events and mortality, reflecting the inherent challenges of hybrid aortic surgery [1, 55].

Multiple studies have compared hybrid repair and conventional open repair in the treatment of aortic arch disease. Ribeiro et al. [55] recently made a comprehensive review comparing the two surgical techniques and found that both have similar outcomes in terms of postoperative mortality and acute neurological events; however, hybrid repair showed fewer respiratory complications. The authors concluded that hybrid repair may be appropriate for patients with high or very high surgical risk, offering a less invasive alternative to conventional open procedures. However, in a long-term perspective, conventional open repair showed more satisfactory results with better reintervention, survival, morbidity, and mortality rates. As such, conventional open repair remains the gold standard for patients who are suitable candidates for major open aortic arch surgery [53, 55].

### Diagnostic imaging

Imaging plays a central role in the diagnosis, planning, and follow-up of aortic arch disease. In most cases, computed tomography angiography (CTA) is the first-line modality due to its wide availability, rapid acquisition, and high-resolution detail. CTA provides critical information about the size and extent of aneurysms or dissections and allows for precise mapping of branch vessel involvement. In both emergency and elective settings, it guides operative planning by helping determine graft dimensions, identifying suitable landing zones for endovascular repair, and informing the decision between open, hybrid, or fully endovascular approaches [61–64]. CTA is also indispensable in postoperative surveillance, where it is used to detect complications such as endoleaks, pseudoaneurysms, or graft migration [65].

Magnetic resonance imaging (MRI), though less commonly used, remains valuable in selected patients, particularly those with contraindications to iodinated contrast or repeated radiation exposure. MRI offers excellent soft tissue resolution and functional flow imaging, but its longer acquisition time and limited availability in emergencies reduce its practicality in acute settings [61].

Intraoperative and bedside imaging modalities also play a key role. Transesophageal echocardiography (TEE) is frequently employed in the operating room and intensive care unit to assess valve function, detect pericardial effusion, or confirm the presence of a dissection flap in real time [66]. Catheter-based angiography is less commonly used for diagnosis but remains valuable in complex or reoperative cases, particularly when high-resolution vascular mapping is required during interventions [67]. Ultimately, the choice of imaging modality depends on the clinical context, patient-specific factors, and the phase of care—whether initial diagnosis, operative planning, or postoperative surveillance.

### Perioperative, intraoperative, and postoperative management

Aortic arch surgery is a highly complex procedure requiring extensive perioperative management, including preoperative, intraoperative, and postoperative care. Preoperatively, patients undergo a comprehensive series of diagnostic scans, such as cardiac catheterisation, echocardiography, carotid duplex scanning, pulmonary function tests, and CT imaging, to assess the anatomy and condition of the aorta, as well as overall surgical fitness. Intraoperative monitoring is critical due to the intricate nature of the surgery and includes central venous pressure (CVP) monitoring, pulmonary artery catheterisation, TEE, and invasive blood pressure measurement at multiple sites (proximal and distal) [68]. This allows for real-time detection of hypoperfusion, which is essential given the high risk of cerebral ischemia. Cerebral blood flow is continuously monitored using non-invasive near-infrared spectroscopy (NIRS), which tracks cerebral tissue oxygenation in real-time, a key factor shown to correlate with neurocognitive outcomes and mortality [69, 70]. Postoperatively, precise blood pressure management is vital to preventing ischemic complications of the brain and spinal cord, with some institutions continuing NIRS monitoring alongside mean arterial pressure, aiming for values above 70 mmHg. Parameters such as hematocrit, oxygenation, and CVP are also closely monitored and optimized to facilitate recovery. Following discharge, patients undergo regular clinical follow-up, including imaging to monitor for complications. In cases involving aortic root diseases, echocardiography is incorporated into the follow-up regimen [14]. As the patient stabilizes, follow-up frequency decreases based on institutional guidelines. Additionally, postoperative neuropsychological testing is often conducted, focusing on cognitive functions, memory, language, and motor skills, given the heightened risk of neurological complications in aortic arch surgery, allowing for the early detection of subtle neurocognitive decline [71].

## Neurological complications: incidence and risk factors

### Overall incidence

The incidence of neurological complications following aortic arch surgery varies across studies but remains a significant concern. In a cohort of 223 patients, PND occurred in 10.3% of cases, which aligns with the broader literature indicating postoperative cerebrovascular events in 10–30% of cases [72]. A larger study analyzing 938 patients over two decades found an overall neurological complication rate of 18.7%, with PND occurring in 6.4% of cases and TND in 12.3% [73]. This study also revealed important long-term outcomes, with late PND occurring in 4.2% of survivors, showing a progressive increase from 2% at 3 years to 6% at 10 years post-surgery [17]. Another significant study reported a total incidence of cerebral complications of 26.72%, with 20.68% being TND and 6.04% being PND [17].

### Patient-related risk factors

Several patient-related characteristics significantly influence the risk of neurological complications. Advanced age has emerged as a crucial risk factor, with studies showing higher median age (71.8 years vs. 67.7 years) in patients who developed complications, attributed to increased atherosclerosis severity [17, 74]. Gender differences have been observed, with some studies indicating higher risk in women (32.6% vs. 27.1%) [74], while others found male patients experienced higher rates of both neurological complications (7.0% vs. 3.3%) and postoperative delirium (14.9% vs. 7.3%) [75].

Pre-existing cardiovascular conditions play a substantial role in risk assessment. Systemic arterial hypertension was present in 68.6% of patients with complications (OR 1.19, 95% CI 1.14–1.24) [74]. Left ventricular dysfunction, particularly poor ejection fraction (< 30%), carried a significant risk (OR 1.48, 95% CI 1.37–1.60), and preoperative atrial fibrillation emerged as a notable risk factor (OR 1.61, 95% CI 1.52–1.70) [74]. A history of cerebrovascular accidents stood out as one of the strongest predictors (OR 2.32, 95% CI 2.16–2.50) [74], with studies showing significantly higher incidence of postoperative PND in patients with previous cerebrovascular accidents (30.8% vs. 9.0%) [72].

Additional risk factors include coronary artery disease, which showed significantly higher risk of complications (*p* = 0.03) [76], and extracardiac arteriopathy (OR 1.94, 95% CI 1.85–2.04) [74]. The underlying etiology of aortic pathology was predominantly atherosclerosis or degenerative changes (67%), followed by acute dissection (30%), and Marfan disease (3%) [76]. Comorbidities such as diabetes mellitus, particularly insulin-dependent, showed increased risk (OR 1.12, 95% CI 1.03–1.21), though interestingly, BMI and smoking status did not emerge as major risk factors for neurological complications [74].

### Procedure-related risk factors

Several surgical and perioperative factors significantly influence neurological outcomes. The type of surgical approach plays a crucial role, with TAR showing significantly higher rates of postoperative neurological deficits compared to other procedures (26.1% vs. 8.5%, OR 3.79) [72]. The choice of cerebral perfusion technique has proven important, with bilateral antegrade cerebral perfusion (b-ACP) demonstrating superior outcomes compared to unilateral perfusion (u-ACP), showing lower rates of both permanent (8.1% vs. 17.7%) and temporary neurologic dysfunction (15.7% vs. 25.8%) [77].

The duration of DHCA is critical, with arrest times exceeding 30 min associated with cognitive impairment, and times over 40 min correlating with increased stroke rates. Survival rates decrease significantly when cerebral ischemia exceeds 60 min [78]. Temperature management during surgery is crucial, with some studies advocating for profound hypothermia (11 °C to 14 °C esophageal temperature) for maximal cerebral metabolic suppression [78].

CPB duration emerged as an independent risk factor (OR = 3.21) [77], and the choice of cannulation site appears significant. Right subclavian/axillary artery cannulation combined with sACP (Selective Antegrade Cerebral Perfusion) under moderate hypothermia (28 °C) showed improved outcomes compared to other approaches [79]. The location of distal anastomosis may also impact risk, with current European guidelines recommending “proximalization” from Zone 3 to Zone 2 to reduce lower body circulatory arrest time [79].

Temperature management strategies have evolved, with studies showing that b-ACP allows for higher arrest temperatures without compromising cerebral protection, potentially making moderate hypothermia combined with appropriate antegrade cerebral perfusion (ACP) technique preferable to DHCA in suitable cases [77]. Additionally, monitoring jugular bulb oxygen saturation until it exceeds 95% before implementing circulatory arrest has been suggested as an important strategy for ensuring maximal metabolic suppression [78].

Overall, the data consistently show that older patients with previous strokes face the highest risk of neurological complications. From a surgical standpoint, total arch replacement carries significantly more risk than simpler procedures, and keeping circulatory arrest times under 30 min appears crucial. The choice of perfusion technique matters too, as bilateral cerebral perfusion clearly outperforms unilateral approaches. These patterns suggest that careful patient selection and meticulous surgical technique can meaningfully reduce complications.

## Pathophysiology of neurological complications

The pathogenesis of neurological complications following aortic arch surgery involves complex and multifactorial mechanisms. These include cerebral hypoperfusion, embolic events, systemic inflammatory responses, and reperfusion Injury, all of which contribute to perioperative neurological injury. A clear understanding of these processes is crutial to minimizing risk.

### Cerebral hypoperfusion

Cerebral hypoperfusion during cardiac surgery is a complex, multifactorial complication influenced by several factors, including temperature, cerebral blood flow, perfusion techniques, pharmacological neuroprotection, aortic clamping, and the duration of DHCA [80, 81]. Cerebral blood flow is typically autoregulated within a specific range of blood pressure values, maintaining constant flow between 50 and 150 mmHg of systolic pressure through local mechanisms that modulate vascular tone [4]. When systolic pressure falls below 50 mmHg, autoregulation is compromised, leading to cerebral hypoperfusion [82]. At normal body temperature, cerebral blood flow averages 55 mL per 100 g of brain tissue per minute. A decline in cerebral blood flow below this range can result in oligemia (between 55 and 20 mL/100 g/min) or frank ischemia (below 20 mL/100 g/min), leading to neuronal dysfunction. When flow drops below 10 mL/100 g/min, there is a release of electrolytes and subsequent neuronal death [83]. Tolerance to cerebral ischemia is enhanced by hypothermia. At normal body temperatures (36.1–37.0 °C), the brain’s oxygen consumption is approximately 2.9 mL/g/min. This consumption is significantly reduced at lower temperatures, dropping to 0.9 mL/g/min at 25 °C and 0.2 mL/g/min at 20 °C [83].

**Fig. 2:**
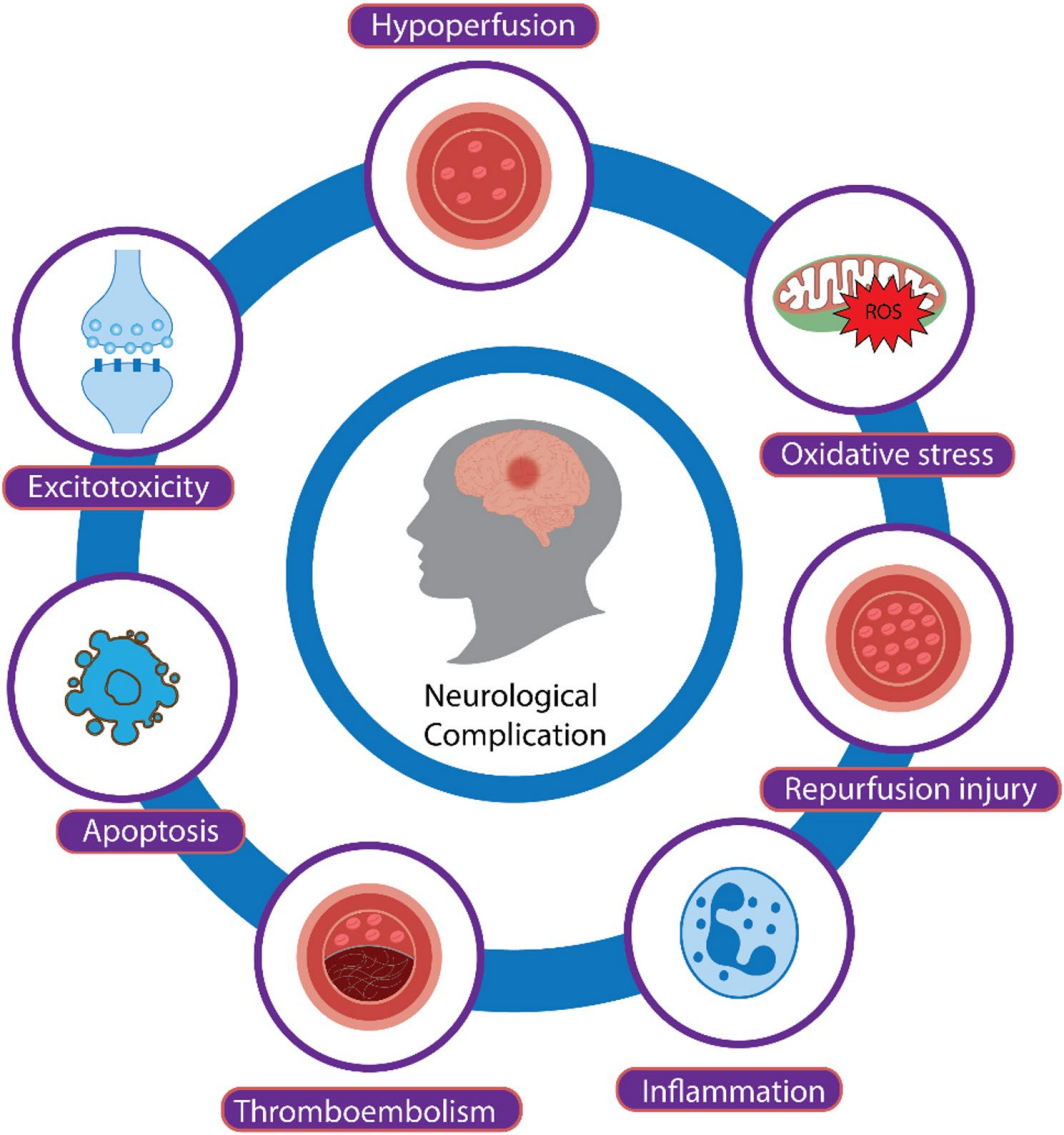
General Mechanisms Leading to Neurological Complications During Aortic Arch Surgery

Aortic arch surgeries often need a circulatory arrest to provide a bloodless surgical field, minimize massive hemorrhage, and reduce cerebral oxygen consumption. This approach interrupts the normal arterial blood supply to the brain, necessitating the use of cerebral perfusion strategies to prevent hypoperfusion and ischemia. However, these techniques may sometimes be insufficient to completely prevent neurological damage [84] (Fig. 2).

Cerebral ischemia induces neuronal injury by triggering the ischemic cascade, which leads to local depletion of oxygen and glucose, impairing the production of high-energy phosphate compounds such as adenosine triphosphate (ATP). This impairment affects essential energy-dependent processes required for cell survival. Neurons, which require a continuous supply of glucose and oxygen to function properly, are particularly vulnerable to hypoxic conditions [85, 86]. The primary mechanisms involved in ischemic neuronal injury include:

I. Excitotoxicity: Reduced cerebral blood flow disrupts ATP production, leading to the failure of ion gradients across neuronal membranes, including those mediated by calcium ATPase, sodium-calcium exchangers, and sodium-potassium ATPase. This disruption causes an excessive influx of calcium into neurons, activating calcium-dependent enzymes that promote the over-release of glutamate and reduce its reuptake. This process results in excitotoxicity, where overstimulation of N-methyl-D-aspartate receptors on postsynaptic neurons generates reactive oxygen species (ROS), causing oxidative stress, mitochondrial dysfunction, and ultimately neuronal death. Additionally, excessive N-methyl-D-aspartate receptor activation can impair neuronal plasticity, adversely affecting memory and learning [86, 87].
II. Oxidative stress: Cerebral hypoperfusion disrupts calcium homeostasis, leading to calcium release within the brain and activating pathways that generate ROS, contributing to oxidative damage [86, 88, 89].
III. Apoptosis: Neuronal apoptosis involves a series of intrinsic and extrinsic pathways that result in neuronal shrinkage and cytoplasmic condensation, culminating in the rupture of the nuclear membrane and the formation of apoptotic bodies. Nutrient and oxygen deprivation interfere with ATP production, shifting metabolism toward anaerobic pathways that produce inadequate ATP to sustain cellular functions [90]. In the Intrinsic Pathway, an ionic imbalance (increased Na+/Ca2 + influx and K + efflux) leads to calcium accumulation within neurons, triggering the excessive release of excitatory neurotransmitters like glutamate. This initiates a cascade of cytotoxic events, including calpain activation, ROS generation, and DNA damage [91]. The extrinsic pathway can operate independently or synergistically with the intrinsic pathway. It is activated by inflammatory signaling factors released by astrocytes, microglia, and oligodendrocytes in response to cerebrovascular injury. These factors include proinflammatory cytokines and receptors such as TNF-α/β, interleukin 1β, and FasL, which activate caspase-8, leading to the activation of downstream effectors such as caspase-3 or BID, mediating apoptosis via a mitochondria-dependent pathway [91].
IV. Neuroinflammation: Following necrosis and apoptosis induced by hypoperfusion, a controlled inflammatory response is initiated, involving ROS, chemokines, and cytokines. This process engages multiple cell types, including innate immune cells (microglia) and adaptive immune cells (lymphocytes) [86, 92].

### Embolism

Emboli are the most common cause of neurological complications following cardiac surgery. Some studies have found that 44% of strokes after cardiac injury were due to embolism [93]. The elevated risk of cerebral embolism during these procedures is primarily attributed to specific surgical maneuvers, such as aortic manipulation, cannulation, and both cross-clamping and side-clamping of the aorta [94]. Additionally, there is a strong correlation between aortic atherosclerosis and the occurrence of cerebral embolisms in cardiac surgeries [95, 96].

On the contrary, microembolisms encompass both air embolism and atheromatous debris. Air embolism occurs especially during mitral valve surgery, where the need to open large cavities of the left side of the heart increases the likelihood of air entrainment [93]. Atheromatous debris from diseased aortic walls is even a greater threat, as proximal aortic atheroma is the strongest independent predictor of stroke with an odds ratio of 4.52 [94]. The risk is especially high with atherosclerotic plaques measuring 4 mm or more in thickness, which are linked to a 4 times higher stroke risk [94]. Vulnerable plaques marked by increased hypoechoicity, calcification, ulceration, and especially mobile components are high-risk lesions linked to a several-fold increase in stroke risk compared to stable plaques [94]. Stroke risk also depends on plaque location, with those in the distal ascending aorta and lesser curvature carrying up to a risk five times higher than plaques in the proximal or anterior ascending aorta [94].

Watershed infarctions represent another critical mechanism occurring at the border zones between major arterial territories, where perfusion pressure is lowest. These infarctions typically result from hypoperfusion episodes combined with microembolism, affecting 12% of patients with postoperative stroke in cardiac surgery series [93]. The anterior circulation is affected in 50% of postoperative strokes, while the posterior circulation accounts for 19%, and watershed territories comprise 12% of cases [93].

Postoperative inflammation and the resulting hypercoagulable states are also significant contributors to embolus formation. When an embolus travels to the cerebral vasculature, it impedes cerebral perfusion, initiating the same pathophysiological cascade as cerebral hypoperfusion, previously described [97].

### Inflammatory response

The inflammatory response following aortic arch surgery can be triggered by several factors, including the surgical procedure itself, ischemia, emboli, or the body’s systemic reaction to trauma [98].

I. Ischemia-Induced Inflammation: The inflammatory response to ischemia can be categorized into three distinct phases: acute, subacute, and chronic. The acute phase occurs within the first few hours following ischemia. During this time, microglia and macrophages begin to clear necrotic cells, and immune cells, particularly neutrophils, start to infiltrate the site of injury. Concurrently, there is a failure of ATPase activity, cellular depolarization, and the onset of excitotoxicity, as described in the hypoperfusion Sect [99].

The subacute phase spans from the initial hours to several days post-ischemia. During this period, the inflammatory process begins to resolve. There is a marked increase in the production of reactive oxygen species (ROS), apoptosis, expression of adhesion molecules, activation of microglia, and infiltration of leukocytes into the brain. Additionally, pro-inflammatory mediators are released, and there is enhanced proteolytic activity along with damage to the blood-brain barrier and endothelial cells [99].

Chronic Phase: Occurring days to weeks after ischemia, this phase is characterized by the release of trophic factors and the initiation of neurogenesis, gliogenesis, and synaptogenesis [99]. Necrotic neurons release damage-associated molecular patterns (DAMPs), which activate toll-like receptor 4 (TLR4) on microglia, prompting them to produce inflammatory cytokines [100, 101]. M1-polarized microglia release cytokines such as IL-1β, IL-6, IL-18, and TNF-α. Perivascular macrophages, located between the brain surface and vascular endothelial membrane, produce IL-1β, IL-12, IL-23, TNF-α, chemokines, and ROS [102, 103].

Upon reaching the brain parenchyma, T helper cells secrete ROS, IFN-γ, TNF-α, IL-1β, IL-17, and IL-21, leading to neuronal and neurovascular damage [104]. Natural killer T cells exert neurotoxic effects by releasing IL-2 and TNF-α [103].

Leukocytes infiltrating the brain parenchyma after ischemia can exacerbate brain injury through various mechanisms. When leukocytes adhere to the endothelium, they may obstruct erythrocytes’ entry into the microvasculature, causing the “no-reflow” phenomenon [105]. Additionally, activated leukocytes can produce ROS, proteases, and matrix metalloproteinases (MMPs), which can damage cerebral blood vessels [106]. Leukocytes also contribute to brain injury by generating phospholipases that produce prostaglandins, leukotrienes, and eicosanoids, leading to vasoconstriction and platelet aggregation. Furthermore, leukocytes can release proinflammatory factors, further exacerbating neuronal damage [107].

II. Systemic Inflammation: Cardiac surgeries are associated with a systemic inflammatory response. Triggers for this response include tissue injury, ischemia, neurohumoral activation, and the subsequent release of coagulation factors, IL-1, IL-6, TNF-α, and the recruitment of leukocytes [108]. Pro-inflammatory cytokines released during systemic inflammation can increase the permeability of the blood-brain barrier, allowing pro-inflammatory molecules and cells to enter the cerebral circulation. This infiltration leads to direct inflammation within the brain, activating microglia and initiating a pro-inflammatory cascade similar to that previously described [108, 109].

### Reperfusion injury

As previously mentioned, aortic arch surgeries require periods of ischemia, either through DHCA or sACP. These periods of controlled ischemia mean that there is no regular blood flow in the body for extended periods. When normal blood flow is restored, the sudden influx of oxygen can lead to the production of reactive oxygen species (ROS), which can cause damage to the central nervous system (CNS) [99].

Following prolonged ischemia, there are metabolic and ultrastructural changes within cells, including altered membrane potentials, ion distribution, cellular inflammation, cytoskeletal disorganization, increased hypoxanthine levels, decreased ATP and phosphocreatine levels, and cellular acidosis. The catabolism of adenine nucleotides during ischemia results in the intracellular accumulation of hypoxanthine, which converts to ROS upon reintroduction of oxygen [110, 111].

Upon reperfusion, there is a significant increase in ROS, including superoxide anions and hydroxyl radicals. These ROS can cause extensive damage to cellular components, including lipids, proteins, and DNA, leading to cellular necrosis through mechanisms such as lipid peroxidation and mitochondrial dysfunction [111].

Reperfusion can also exacerbate the inflammatory response, characterized by the activation of neutrophils, the complement system, and the release of pro-inflammatory cytokines. This inflammatory cascade can cause local damage or extend to remote organs, potentially leading to systemic inflammatory response syndrome (SIRS) or multiple organ dysfunction syndrome (MODS) [110].

Additionally, during ischemia, ion homeostasis is disrupted, specifically with increased intracellular sodium and calcium levels due to the failure of the Na+/K+/ATPase pump. Upon reperfusion, the sudden influx of these ions can lead to cytotoxic edema and cellular necrosis [110, 112]. Reperfusion can also trigger a mitochondrial permeability transition, which can result in the release of pro-apoptotic factors, such as cytochrome c, into the cytoplasm, promoting cell death [113].

## Types of neurological complications

### Stroke

**Fig. 3:**
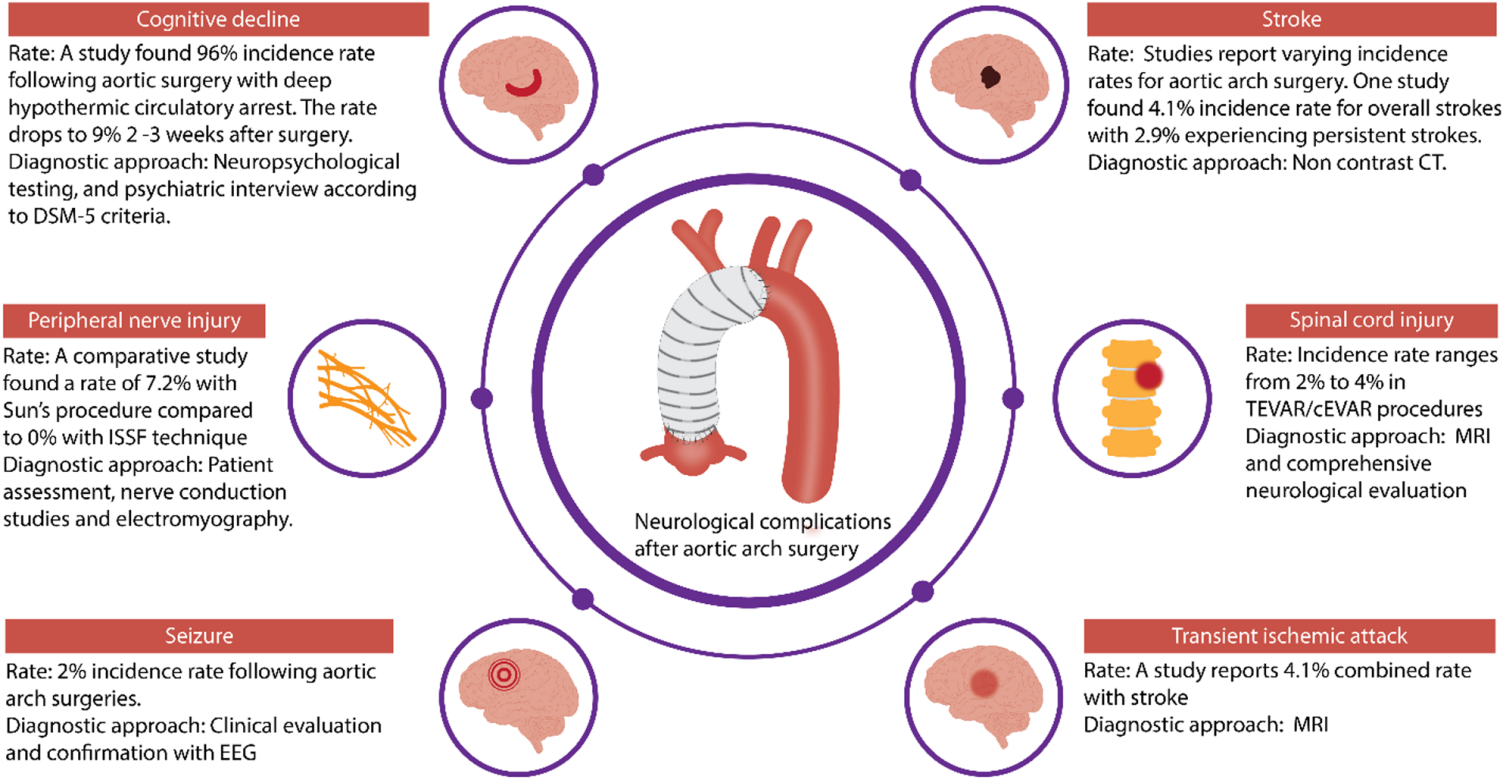
Summary Figure of Neurological Complications Associated with Aortic Arch Surgery

Stroke remains one of the most significant complications following aortic arch surgery, with studies reporting varying incidence rates. In one large cohort study of 938 patients undergoing elective aortic arch surgery, the overall stroke rate was 4.1% (Fig. 3), with 2.9% experiencing persistent stroke [114]. The majority of strokes (25 out of 27) were embolic in nature, with only two being hemorrhagic [114]. Research has identified two main mechanisms for stroke occurrence: embolic phenomena and hypoperfusion. Embolic strokes can result from debris from atherosclerotic brachiocephalic vessels or from surgical instrumentation, while hypoperfusion-related strokes can occur due to malperfusion from static or dynamic obstructive dissection flaps impacting the carotids, or from inadequate cerebral protection during circulatory arrest [115]. When analyzing different cannulation techniques, persistent stroke rates were 3.5% for axillary cannulation and 1.9% for innominate cannulation, though this difference was not statistically significant [114]. The distribution pattern of embolic strokes varied, with 7 having right-sided distribution, 10 left-sided, and 6 bilateral distribution [114]. Three independent predictors of persistent stroke have been identified: previous history of cerebrovascular disease, previous renal history, and antegrade cerebral perfusion time greater than 30 min [114].

### Transient ischemic attack (TIA)

The available literature provides limited specific data on TIAs as a distinct entity. One study reported combined stroke/TIA rates of 4.1% in patients without significant intraoperative neurophysiologic monitoring (IONM) changes and 10.9% in those with IONM changes [115]. However, the study did not differentiate between strokes and TIAs in their detailed analysis, focusing instead on the timing of events (early vs. late) rather than the type of neurological event [115].

### SCI

**Fig. 4:**
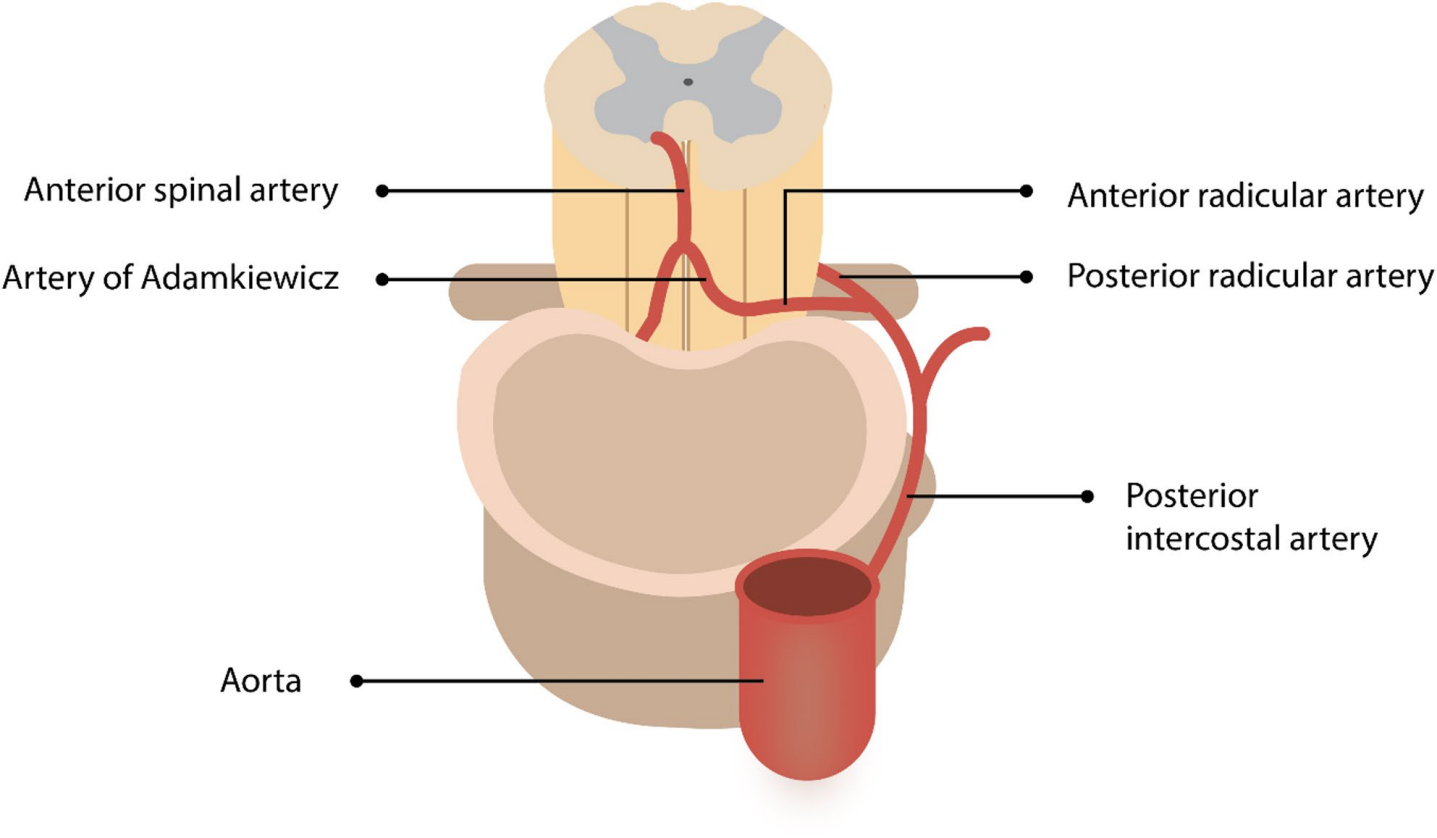
Schematic illustrating spinal cord vascular anatomy

SCI represents a devastating complication of aortic surgery, with reported incidence rates ranging from 2% to 11% in TEVAR/cEVAR procedures [116]. The risk increases substantially after just 30 min of spinal cord ischemia during aneurysm repair [117]. Type II thoracoabdominal aortic aneurysm patients face the highest risk, with historical rates of approximately 31% (increasing to 33% with dissection) when using the simple cross-clamp technique without protective measures [117]. Spinal cord perfusion depends primarily on the anterior spinal artery (ASA), which supplies the anterior two-thirds of the cord, including critical motor pathways. One of the most important contributors to the ASA is the artery of Adamkiewicz (Fig. 4), also known as the great anterior radiculomedullary artery [118]. The artery in question most often originates between the T8 and L2 vertebral levels, commonly branching from a left-sided posterior intercostal artery. It serves as the primary source of blood flow to the thoracolumbar portion of the spinal cord—an area particularly susceptible to ischemic injury during surgeries involving the descending aorta [118, 119]. If the artery of Adamkiewicz or its associated segmental feeders are disrupted during such procedures, perfusion to the ASA can drop sharply, leading to a significantly increased risk of paraplegia [120]. The threat is even greater in individuals with limited collateral circulation or atypical vascular patterns [121]. To mitigate this, detailed imaging studies like CT or MR angiography are often performed before surgery to locate the Adamkiewicz artery and assist in planning [122, 123]. This level of planning becomes especially critical in complex surgeries such as FET procedures or extensive thoracoabdominal repairs, where lower thoracic segmental arteries are frequently at risk [124]. The implementation of protective surgical adjuncts, specifically cerebrospinal fluid drainage (CSFD) and distal aortic perfusion, has significantly improved outcomes, reducing neurological complications from 19% to 9% in comparative studies [117]. Recent data shows encouraging trends, with SCI rates declining from 4.5% in 2014 to 1.4% in 2018 [116]. Notably, patients receiving preoperative prophylactic CSFD showed better outcomes compared to those receiving postoperative therapeutic CSFD, with only 54% versus 79% experiencing permanent paraplegia at discharge, respectively [116].

### Neurocognitive dysfunction

Evidence has shown that new neurocognitive dysfunction can occur post-aortic arch surgery. The incidence was noted to be around 96% in patients 3 to 6 days after they underwent aortic arch surgery with the use of DHCA. This big proportion of new impairment drops to 9% at 2 to 3 weeks after surgery [125]. Postoperative cognitive dysfunction (POCD) and delirium are two forms of neurocognitive dysfunction of significant concern following aortic surgery. POCD’s exact mechanisms remain unclear but are likely multifactorial, with cerebral micro- and macro-embolism playing a significant role [126]. Pre-existing cerebral vascular disease contributes to limited cognitive reserve, making patients particularly vulnerable to brain hypoperfusion during surgery [126]. Delirium affects approximately 35% of patients and is associated with extended ICU care, increased risk of complications, and long-term cognitive decline [127]. Studies have identified specific biological markers (TR4 and EZH2) that are elevated before surgery in patients who later develop delirium [127]. Interestingly, hallucinations were observed in 39% of patients and can occur independently of delirium, with distinct biological markers differentiating between these cognitive disturbances [127].

Although many patients with neurocognitive dysfunctions regain their cognitive abilities within weeks or months after surgery, some continue to experience subtle yet lasting impairments over time [128]. Notably, a pivotal study revealed that individuals who showed signs of POCD shortly after surgery were more likely to experience cognitive decline years later—specifically between five and seven years post-operation—and had a higher risk of mortality within the first year, especially among older adults [129]. These enduring issues can affect key areas such as memory, executive functioning, and overall quality of life [130]. As a result, high-risk patients must undergo ongoing monitoring, including regular cognitive evaluations and imaging studies, to catch any signs of progressive deterioration early [131].

### Seizures

Seizures are a well-documented though relatively uncommon complication following cardiac surgery, with incidence ranging from 0.5% to 7.6% [132]. In a large study of 7,280 cardiac surgery patients, 0.8% experienced postoperative seizures, with 59% of these cases developing recurrent seizures, mostly within 24 h of the first event [132]. The majority (81%) manifested as grand mal seizures, while 8% developed into non-convulsive status epilepticus [132]. In patients receiving venoatrial extracorporeal membrane oxygenation support, seizures occurred in 3.4% of cases, making them the third most common neurological complication after cerebral infarction (8.0%) and brain death (7.2%) [133]. Seizures following aortic arch surgery are particularly associated with a higher incidence compared to other cardiac procedures [134]. It is estimated that 2% of aortic arch surgery patients experience clinically evident seizures, and around 0.9% present with electrographic seizures [134]. Risk factors for recurrent seizures include elevated preoperative creatinine levels, procedures involving the thoracic aorta, and early seizure onset [132]. Hyperlipidemia has been independently associated with seizure occurrence during VA-ECMO support [133].

Seizures following aortic arch surgery often develop due to a combination of factors, rather than a single identifiable cause. One of the major causes are small emboli that can be released during the operation. These might come from manipulating the aorta or from using the heart-lung machine. Emboli can carry bits of plaque, clots, or even tiny air bubbles into the brain’s blood vessels. This then disrupts local blood flow and irritates the brain’s surface, both of which can make seizures more likely [135]. Studies using imaging tools like MRI and transcranial Doppler have shown that many of these emboli don’t cause obvious symptoms but are still linked to changes in brain waves and seizures later on [136, 137].

Another contributing factor is poor blood flow to the brain during surgery. If blood pressure drops too low, or if the brain is unable to regulate its own blood supply under stress properly, neurons may not receive an adequate oxygen and nutrient supply. This disruption could interfere with how ions move across membranes and make inhibitory signals less effective, setting the stage for abnormal electrical activity [138]. Some parts of the brain, such as the hippocampus, are especially prone to this kind of stress and may be the first to exhibit dysfunction.

Even after blood flow is restored, the brain may still be at risk. Reperfusion can induce secondary injury through a cascade of biochemical changes, including excessive glutamate release and oxidative stress. These events can compromise the integrity of the blood-brain barrier and make the brain more susceptible to seizures [139]. Additionally, pro-inflammatory molecules like IL-1β and TNF-α are often upregulated in this context and are known to contribute to seizure activity [140].

### Peripheral nerve injuries

Peripheral neuropathies are rare but debilitating neurological complications following cardiac procedures in general. Common neuropathies include vagus nerve, recurrent laryngeal nerve, phrenic nerve, and brachial plexus, other peripheral nerves, as well as visual pathways neuropathies [141]. While there is limited data on the incidence rate following aortic surgery, studies indicate that the rates vary by surgical technique. A comparative study showed a 7.2% incidence rate with the conventional Sun’s procedure compared to 0% with the new in-situ stent-graft fenestration (ISSF) technique [142]. The pathophysiology is often multifactorial, including ischemia, stretch, local compression, and metabolic abnormalities. These complications range from mild to serious and debilitating outcomes [141].

A clear example of how surgical technique can contribute to these mechanisms is median sternotomy, which is commonly used to access the heart and major vessels. This procedure can sometimes put the brachial plexus at risk, especially if the chest is opened too wide or pulled with too much lateral force [143]. Also, the use of sternal retractors over long periods, or with excessive pressure, may unintentionally compress nearby nerves like the phrenic or recurrent laryngeal. This risk tends to go up in reoperations or when anatomical changes are present [144].

Patient positioning is another crucial factor. Overstretching of the arms, or hyperextension of the shoulders, or sharp head movements in one direction for prolonged periods can increase the risk of nerve stretch or entrapment. This is especially true for nerves like the ulnar, radial, and parts of the brachial plexus [145]. Such injuries are often preventable via the utilization of simple interventions such as adequate padding, proper arm support, maintaining neutral limb and head position, and periodically reassessing patient positioning during prolonged surgeries [146].

## Prevention strategies

### Preoperative assessment and optimization

Preoperative risk assessment and optimization play a crucial role in preventing neurological complications after aortic arch surgery. Low preoperative fibrinogen levels (< 3.425 g/L) have been identified as a risk factor for neurological complications [147]. Other preoperative factors associated with increased risk include elevated serum creatinine levels and higher white blood cell counts [147]. A history of stroke is the strongest predictor of adverse outcomes, with an odds ratio of 7.846 [148]. Preoperative neurological deficits, such as hemiparesis, TIA, syncope, and disturbed consciousness, are strongly associated with transient neurological dysfunction postoperatively [148]. Emergency surgery and hemodynamic instability are also independent predictors of adverse outcomes [148]. These findings underscore the importance of thorough preoperative neurological assessment, optimization of fibrinogen levels, renal function, and inflammatory markers, as well as hemodynamic stabilization when possible [147, 148].

### Intraoperative neuroprotection techniques

#### Cerebral perfusion strategies

ACP and retrograde cerebral perfusion (RCP) are two well-established cerebral perfusion strategies utilized by surgeons to mitigate the risk of neurological injuries during aortic arch surgery [149]. ACP involves the supply of blood in a physiological direction (antegrade) to the brain through cerebral arteries. Access is mainly achieved through cannulation of the axillary, innominate, or carotid arteries [149, 150]. Depending on factors such as patient anatomy and duration of circulatory arrest, the perfusion could be performed either unilaterally or bilaterally [151]. sACP is a more precise term often used interchangeably with ACP. It emphasizes that perfusion is targeted exclusively to the brain, while the rest of the body remains under circulatory arrest during the procedure.

Among the available cannulation sites for cerebral perfusion, the right axillary artery has emerged as the preferred site in many centers, largely due to its association with better neurological outcomes and its compatibility with sACP. It allows for dependable flow and supports both unilateral and bilateral cerebral perfusion, all while avoiding direct interference with the arch itself [152]. In recent years, the innominate artery has also gained traction, particularly in elective cases. It offers a more central cannulation approach and has shown promising results when used under controlled conditions. On the other hand, the femoral artery, though less favored, remains useful in certain scenarios—especially when access to the upper body vessels is limited or when retrograde perfusion is considered appropriate [153]. The 2023 EACTS/STS guidelines reinforce these trends, clearly recommending the use of axillary or innominate artery cannulation in arch procedures requiring circulatory arrest. These approaches are especially emphasized in situations where protecting cerebral function is a top priority [49].

In contrast to ACP, RCP involves the supply of blood in the reverse direction (retrograde) through the cerebral venous system, most often through the superior vena cava [150, 154]. This method allows surgeons to flush air and particulate emboli from circulation while providing some degree of oxygen delivery during circulatory arrest [154, 155]. Numerous studies have compared both ACP and RCP in terms of neurologic outcomes, mortality, and postoperative recovery metrics (Table 2). Okita et al. analyzed 8,169 patients who underwent total arch replacement from a nationwide Japanese database, comparing outcomes between ACP and RCP. A propensity score analysis was performed matching 1,141 RCP patients with ACP patients. They found no significant difference between ACP and RCP in terms of PND (6.7% vs. 8.6%), TND (4.1% vs. 4.4%), or mortality (3.2% vs. 4.0%), although the ACP group had a shorter stay in the ICU (>8 days: 15.6% vs. 24.2%) [156]. Consistently, Englum et al., utilizing data from the Society of Thoracic Surgeons database, conducted a comparative study of aortic arch repairs involving 4,418 cases with ACP and 3,149 with RCP. There was no significant difference in neurological complications and postoperative mortality between the two adjuncts [157].

These findings have been reinforced by a network meta-analysis encompassing 26,968 patients. In the study by Hameed et al., both ACP and RCP were associated with reduced rates of primary outcomes (postoperative stroke and operative mortality) compared to DHCA alone. ACP significantly reduced stroke (OR 0.62, 95% CI 0.51–0.75) and mortality (OR 0.63, 95% CI 0.51–0.76), while RCP also showed reductions in stroke (OR 0.66, 95% CI 0.54–0.82) and mortality (OR 0.57, 95% CI 0.45–0.71), however, no significant difference was observed between the two techniques in terms of primary and secondary outcomes (postoperative TND, respiratory complications, and renal failure) [158].

Overall, while both ACP and RCP are clinically effective, current evidence does not establish the superiority of one technique over the other. Nevertheless, ACP is generally preferred by surgeons due to its physiologic flow pattern, more uniform cerebral blood flow distribution, favorable clinical outcomes, and the capacity to safely extend circulatory arrest beyond 80 min.

#### Hypothermia

The classification of hypothermia during aortic arch surgery follows established temperature ranges with distinct clinical applications. According to international consensus guidelines, profound hypothermia (< 14 °C), deep hypothermia (14.1–20 °C), moderate hypothermia (20.1–28 °C), and mild hypothermia (28.1–34 °C) each serve specific roles [159]. Profound hypothermia is rarely employed in contemporary practice due to associated complications.

Hypothermia provides neuroprotection by drastically reducing metabolic rate, decreasing cerebral oxygen consumption by approximately 6–7% for every 1 °C drop in body temperature (from 37 °C to 27 °C) [160, 161]. This theoretical benefit underpinned the earlier use of deep hypothermic circulatory arrest in aortic arch surgery [162]. However, deep hypothermia may have adverse effects, including neuronal injury caused by adenosine triphosphate loss, glutamate receptor activation, and Ca2 + influx. Deep hypothermia also may impair microvascular perfusion and disrupt the blood-brain barrier [160]. The adverse effects of DHCA are linked with worse neurologic and renal outcomes, such as postoperative stroke and kidney injury. To reduce these complications and achieve improved outcomes, surgical strategies have increasingly shifted toward warmer temperature ranges combined with perfusion techniques such as ACP [162]. In a study by Tsai et al., MHCA (≥ 20 °C) combined with antegrade cerebral perfusion showed superior outcomes compared to deep hypothermic approaches. MHCA was associated with lower hospital and 30-day mortality rates and fewer neurologic sequelae than DHCA. The 30-day mortality rate was 4-fold higher with DHCA, and stroke incidence was nearly threefold higher (8% vs. 3%) [160].

Further exploring the use of warmer temperatures, Zhu et al. demonstrated that mild hypothermic circulatory arrest (around 31 °C) combined with sACP is both safe and effective. This approach demonstrated organ protection comparable to moderate hypothermia strategies while offering additional benefits of shorter operative, CPB, and cross-clamp times. These reductions were accompanied by decreased complications such as coagulopathy and acute kidney injury. Moreover, mild hypothermic circulatory arrest was associated with similar mortality and TND rates compared to moderate hypothermia, but tended to have lower rates of PND [159].

A recent network meta-analysis comparing mild, moderate, and deep hypothermia for circulatory arrest in aortic arch surgery subsequently supports the use of warmer temperature strategies. Compared to deep hypothermia, both mild and moderate hypothermia were found to be associated with reduced mortality (mild vs. deep: OR 0.50; 95% CI 0.29–0.87, moderate vs. deep: OR 0.68; 95% CI 0.54–0.86). Mild hypothermia, in particular, was also associated with significantly lower rates of strokes (OR 0.50; 95% CI 0.28–0.89), AKI (OR 0.36; 95% CI 0.15–0.88), and postoperative bleeding (OR 0.55; 95% CI 0.31–0.97) compared to deep hypothermia. While there was no significant difference between mild and moderate hypothermia in these outcomes, mild hypothermia was associated with shorter operative times and hospital stays [163].

These findings reflect an evolution in surgical techniques and temperature strategies over time. The effectiveness of mild and moderate hypothermia, when combined with appropriate cerebral perfusion, has led to a shift away from deep hypothermia. The clinical applications are as follows: Mild Hypothermia (28.1–34 °C) is suitable for routine arch replacement with anticipated circulatory arrest < 30 min in patients with good neurological status. Moderate Hypothermia (20.1–28 °C) represents the current standard for total arch procedures and moderate surgical complexity. Deep Hypothermia (14.1–20 °C) remains reserved for complex pathology requiring prolonged circulatory arrest or when ACP is not feasible, despite its association with increased coagulopathy risk and extended cooling and rewarming times.

While most evidence is based on adult populations, conflicting results have been reported in pediatric settings. For instance, DHCA showed significantly higher neurological complications compared to moderate hypothermia with sACP [164]. This suggests that optimal temperature management strategy may differ across age groups, and findings in the adult population should not be directly extrapolated in pediatric cases. Apparent contradictions among studies comparing hypothermia protocols for arch surgery may also stem from several factors, including differences in patient selection criteria, temporal changes in clinical practice, variations in surgical techniques employed in conjunction with hypothermia, and inconsistencies in how outcomes are defined and measured.

**Table 2:** Summary table of studies investigating neurological outcomes of various cerebral perfusion and temperature management strategies in aortic arch surgery

Perfusion strategies					
Author [Year]	Study type	Strategy compared	Temperature	Sample size	Outcomes
Englum et al. [2017] [157]	(2017) Comparative effectiveness	ACP, RCP, No CP	Ranges: ≤20 °C (Deep/profound), 20.1–24 °C (low-moderate), 24.1–28 °C (high-moderate)	12,521	Worse outcomes in terms of mortality and stroke in patients without CP
Ganapathi et al. 2014	Propensity matched analysis	ACP vs. RCP	14 °C	160 (80 ACP vs. 80 RCP in matched analysis)	Adjusted comparison revealed no significant difference in rates of neurologic complications or 30-day/in hospital mortality
Okita et al. 2015 [156]	Propensity matched analysis	ACP vs. RCP	21.2 °C ± 3.7 °C vs. 24.2 °C ± 3.2 °C (rectal)	8, 169 patients after exclusions, 2,282 (1,141 ACP vs. 1141 RCP in matche d analysis)	no significant difference in rates of strokes, TND or 30-day mortality
Perreas et al. [2016] [165]	Propensity-matched retrospective analysis	DHCA/RCP vs. MHCA/ACP	18 °C (IQR: 17–20 °C) vs. 23.2 °C (IQR: 21.9–24.6 °C)	80 (40 DHCA/RCP vs. 40 MHCA/ACP)	Neuro complications: 39.2% (DHCA/RCP) vs. 14.3% (MHCA/ACP)
Itagaki et al. [2019] [166]	Multivariable analysis	ACP, RCP, No CP	20–23 °C	7,830	Death/PND: ACP 11%, RCP 8%, No CP 14%
Li et al. [2016] [167]	Propensity analysis	ACP, higher vs. lower perfusion pressure	24–25 °C	800 patients, 51 patients excluded due to lack of data	TND: 7.08%, PND: 7.34%. No difference in rate of neurological complications between high and low pressure.
Kamenskaya et al. [2017] [168]	RCT	DHCA vs. ACP	18 °C vs. 24 °C	58	Higher neuro complications in DHCA (37.9%) vs. ACP (13.8%)

Temperature management					
Author [Year]	Study type	Strategy	Temperature	Sample size	Neuro outcomes
Liu et al. [2017] [148]	Retrospective study	DHCA + SACP	18–20 °C (nasopharyngeal), 22–25 °C (bladder)	626	PND 1.9%, TND 13.9%, 30-day Mortality 4.6%
Zhu et al. [2022] [159]	Retrospective cohort study	Mi-HCA vs. MHCA	25–31 °C	138	Comparable mortality and TND rates, but Mi-HCA tended to have lower rates of PND
Kornilov et al. [2015] [164]	Retrospective study	DHCA vs. MHCA + SACP	18–27 °C	62	Neuro complications: DHCA 30.8% vs. SACP 5.9%
Tsai et al. [2013] [160]	Retrospective study	Deep vs. Moderate Hypothermia (SACP)	< 20 °C vs. ~23 °C	221	Stroke: 8% (deep) vs. 3% (moderate); Mortality was lower in moderate

ACP – Antegrade Cerebral Perfusion, CP – Cerebral Perfusion, DHCA – Deep Hypothermic Circulatory Arrest, HCA – Hypothermic Circulatory Arrest, ICU – Intensive Care Unit, IQR – Interquartile Range, MHCA – Moderate Hypothermic Circulatory Arrest, Mi-HCA – Mild, Hypothermic Circulatory Arrest, PND – Permanent Neurological Dysfunction, RCP – Retrograde Cerebral Perfusion, SACP – Selective Antegrade Cerebral Perfusion, TND – Transient Neurological Dysfunction

#### Pharmacological interventions

Several pharmacological agents have been investigated for their potential neuroprotective effects during aortic arch surgery. Levosimendan, an inotropic agent, showed some promise in lowering intracranial pressure in a porcine model of hypothermic circulatory arrest but did not demonstrate persistent neuroprotective effects or improvements in cerebral metabolism, neurological recovery, or brain histopathology [169]. In a rat model, levosimendan improved cardiac function during rewarming after DHCA compared to epinephrine, potentially supporting better brain perfusion, but neurological endpoints were not directly assessed [170].

Hypertonic saline dextran (HSD) has shown promising results in animal studies, with significantly better neurologic recovery, lower intracranial pressure, higher cerebral perfusion pressure, and better-preserved brain metabolism after hypothermic circulatory arrest [171]. Thiopental has also been found beneficial in several studies, lowering cerebral oxygen consumption and producing an isoelectric EEG (Electroencephalography*)* when used in combination with hypothermia [172]. However, the timing of thiopental administration is crucial, as administering it too early before circulatory arrest may potentially be detrimental [172].

### Monitoring techniques

1. *Cerebral oximetry*

NIRS for monitoring regional cerebral oxygen saturation (rScO2) has gained popularity in aortic arch surgery. A decrease in rScO2 of >20% from baseline or an absolute value of < 50% is often considered clinically significant [126]. These threshold values were derived from studies comparing rScO2 monitoring with other modalities during carotid artery clamping, where decrements >20% from baseline or absolute values < 50% showed 44–100% sensitivity and 44–82% specificity for detecting cerebral ischemia [126]. Based on current evidence, it is recommended to use the 20% decrease from baseline threshold as the primary criterion for intervention, as this has been associated with cognitive decline in multiple studies [126]. NIRS monitoring may allow early detection of CPB cannula malposition and has been associated with lower non-adjusted stroke risk compared to historical controls [126]. Some studies have found links between rScO2 desaturation during CPB and cognitive decline, though there is variability in assessment methods and timing [126].

In aortic arch surgery specifically, NIRS has been used to continuously monitor rSO2 in both cerebral hemispheres during low-flow antegrade selective cerebral perfusion (ASCP) [173]. Sudden decreases in rSO2 below desaturation thresholds correlated with displacement or incorrect positioning of the arterial cannula [173]. While NIRS appears to be a useful technique for detecting perfusion failures and cannula malpositioning, the relationship between specific rSO2 values/thresholds and neurological outcomes remains unclear [173].

Additionally, NIRS has important limitations, including its inability to distinguish arterial from venous blood, with algorithms assuming a fixed distribution of 70–75% venous to 25–30% arterial blood [126]. Additionally, NIRS only monitors the outer layer of the frontal cerebral lobe, potentially missing ischemia in other brain regions [126]. Given these limitations, a multimodal monitoring approach combining NIRS with EEG and jugular bulb oxygen saturation monitoring may provide a more comprehensive neurological assessment during complex aortic procedures.

2. *EEG*

EEG has been used to detect brain ischemia during cardiac surgery, including aortic arch procedures. However, its reliability is limited by many confounding factors, such as anesthesia depth, hypothermia, medications, electromyographic activity, seizures, and electrical interference [126]. EEG only monitors superficial layers of the cerebral cortex, so ischemia in deeper brain regions may go undetected [126]. As a result, neurological complications like stroke can occur without EEG changes, and not all EEG changes necessarily predict postoperative stroke [126].

Processed EEG (pEEG) monitors like BIS have gained popularity due to their simplified interface, but studies have shown mixed results regarding their ability to prevent intraoperative awareness or improve recovery times after cardiac surgery [126]. Some evidence suggests that BIS-guided anesthetic titration may reduce postoperative delirium, but the results are not conclusive [126]. To overcome these individual limitations, combining EEG with other monitoring modalities such as NIRS and jugular bulb venous oxygen saturation may provide complementary information for comprehensive neurological monitoring during high-risk procedures.

3. *Transcranial Doppler (TCD) ultrasonography*

TCD ultrasonography has shown limitations in reliably monitoring cerebral blood flow during low-flow antegrade cerebral perfusion, particularly in neonates undergoing aortic arch surgery [174]. TCD failed to detect a signal in several neonates at various flow rates, with its ability to detect cerebral perfusion decreasing as flow rates were reduced [174]. This makes TCD less ideal as a sole monitoring technique for ensuring adequate cerebral perfusion during these procedures [174].

In summary, a multimodal approach to neuroprotection and monitoring during aortic arch surgery appears to be the most promising strategy. This includes careful preoperative assessment and optimization, the use of sACP with appropriate hypothermia, consideration of pharmacological interventions, and a combination of monitoring techniques to overcome the limitations of individual methods. Further research is needed to refine these strategies and establish their long-term efficacy in reducing neurological complications.

## Management of meurological complications

### Immediate postoperative care

In the immediate postoperative phase, diligent monitoring and neuroprotection are paramount. Blood pressure management is essential, particularly after hybrid aortic arch repair, to avoid cerebral and spinal cord injuries. Cerebral blood flow is commonly assessed through NIRS monitoring, which plays a critical role in the initial postoperative period [175]. Once the patient is admitted to the ICU, the main goals include maintaining hemodynamic stability, providing adequate ventilatory support, addressing coagulopathy, and maintaining normothermia and normoglycemia. Hypotension, often due to hypovolemia, is common during this phase, necessitating close observation of chest tube and drain outputs [42]. Optimal resuscitation with fluids and, if necessary, blood products is critical to managing hypovolemia. As the patient demonstrates stable hemodynamic, respiratory, and metabolic outcomes, sedation should be gradually lightened to allow for a spontaneous breathing trial (SBT) and, when feasible, extubation [42]. Post-extubation, two primary management pathways are typically followed. If the patient shows no signs of end-organ damage, the focus shifts to preventing ICU-related complications such as delirium, infection, malnutrition, and deconditioning, with the ultimate goal of restoring the patient’s preoperative functional status as quickly as possible [42]. However, if perioperative end-organ damage is evident, a targeted approach to diagnostics and treatment for the affected organ systems becomes essential. This scenario often results in an extended ICU and hospital stay, heightening the importance of preventing ICU-related complications. In these cases, comprehensive and proactive management is critical to supporting recovery and minimizing long-term functional impairment [42].

### Diagnostic and treatment approaches for specific complications

#### Stroke

The detection of postoperative stroke can be challenging due to the sedative and analgesic effects of general anaesthesia. To mitigate this, short-acting intravenous sedatives and analgesics (such as dexmedetomidine, propofol, and fentanyl) are recommended in the immediate postoperative period [176]. Non-contrast CT remains the primary imaging modality for stroke diagnosis, with contrast-enhanced CT reserved for certain cases, keeping in mind potential risks such as renal dysfunction from intravenous contrast. A thorough neurological assessment is also advised if any focal neurological deficits are observed​ [176]. In the postoperative setting, stroke management typically includes the administration of aspirin, a statin, and the careful prevention of hyperthermia and hypoglycemia. Due to the elevated risk of bleeding immediately following surgery, anticoagulants are generally contraindicated, and thrombolysis is deemed too risky in this period. However, intra-arterial fibrinolysis might be an alternative, though evidence for its safety in postoperative settings is limited and warrants further research [176]​.

#### SCI

SCI is a severe, life-altering complication that can lead to varying degrees of motor and sensory deficits, from temporary paraparesis to permanent paralysis. SCI presents with new lower extremity motor or sensory deficits not attributable to other causes [177]. An altered postoperative neurological examination compared to baseline is a key indicator of SCI, with diagnosis often confirmed through spinal MRI and a comprehensive neurological evaluation. SCI can lead to a wide range of disabilities, underscoring the need for immediate identification and management [177]​. For SCI, once identified, the patient should be monitored in the cardiothoracic ICU with continuous hemodynamic surveillance and hourly neurological assessments. If a cerebrospinal fluid (CSF) drain is not already in place, it should be inserted promptly, ideally within one hour of SCI detection. CSF drainage is then initiated with a target CSF pressure of ≤ 10 mm Hg, adjusting serially based on neurological recovery. To enhance spinal cord perfusion, additional interventions may include volume resuscitation, vasopressor support, and augmented oxygen delivery [177]​.

#### Neurocognitive decline

The general method of diagnosing delirium is through a formal psychiatric interview according to DSM-5 criteria [178]. Although other tools for assessing delirium exist, studies find them more appropriate for specific patients. For example, the CAM-ICU is more appropriate for detecting delirium in intubated patients, while the 3D-CAM is more appropriate for non-intubated patients. Although these tools are more sensitive than chart review alone, their accuracy can be improved when combined with it, especially to detect delirium that can be missed due to its fluctuating course [178]. POCD is assessed by pre- and postoperative neuropsychological testing of the patient [178]. According to the international POCD nomenclature recommendation, a 1-SD drop in test performance occurring between 30 days and one year is defined as mild, while a 2-SD drop is defined as major POCD. However, these recommendations do not specify which specific cognitive test to use [178]. While POCD is usually a transient, reversible condition occurring shortly after surgery, early dementia—such as Alzheimer’s or vascular dementia—presents as a progressive cognitive decline [179]. Distinguishing between the two can be clinically challenging, particularly in elderly patients or those with pre-existing cerebrovascular disease. POCD often improves over weeks to months. In contrast, dementia is marked by continuous deterioration [180]. Neuropsychological testing, imaging (such as MRI for cortical atrophy or white matter lesions), and longitudinal assessment are vital to accurately differentiate between POCD and dementia [129]. Additionally, the presence of biomarkers (e.g., APOE ε4) may help identify patients at risk of progressive cognitive decline [181]. Currently, there is no definitive treatment strategy for neurocognitive decline, emphasizing that primary prevention remains the most effective approach. Several approaches, such as early postoperative ambulation, vagus nerve stimulation, video game-based brain training, physical exercise, and non-invasive transcranial magnetic and electrical brain stimulation, to diet interventions, have been proposed to improve neurocognitive functions [178]. Further research is essential to better understand the mechanisms underlying POCD, which could facilitate the development of targeted preventive and therapeutic strategies to mitigate its impact [182].

#### Seizure

Clinical evaluation and confirmation with an electroencephalogram (EEG) are key for the diagnosis of seizures​ [134]. The treatment approach for seizures varies depending on the duration [183]. For seizures lasting more than 2–3 min, immediate stabilisation is necessary to prevent the development of status epilepticus. Stabilisation involves administering oxygen and benzodiazepines (e.g., intravenous lorazepam, diazepam, or midazolam), with additional doses if necessary. If the seizure persists despite initial treatment, second-line antiepileptic drugs or continuous intravenous sedation with propofol may be required. Once the patient is stabilised, it is essential to investigate and address any underlying causes to prevent future episodes [183].

#### Peripheral nerve injury

Early diagnosis is imperative for patients with peripheral nerve injuries. This involves patient assessment, such as a full history and examination. Other investigations include nerve conduction studies and electromyography [141]. Electrophysiology studies can also be used to track nerve injury recovery over time. The majority of the time, peripheral nerve injuries may resolve between six and twelve weeks, and more than half of patients recover full sensory and motor functions within a year. Permanent injuries may be experienced by patients with poor recovery. This could be a minor injury, such as a small area of sensory loss, or a major and disabling condition, such as chronic pain and significant upper or lower limb movement disorder, and can have a significant impact on the quality of life (QoL) of patients [141].

#### TIA

The diagnostic criteria and techniques for TIA have advanced significantly in recent years. Current guidelines recommend that patients presenting with TIA undergo neuroimaging evaluation within 24 h of symptom onset, with MRI, including diffusion-weighted sequences, being the preferred modality [184]. Additionally, noninvasive imaging of cervical vessels is essential, while noninvasive imaging of intracranial vessels is strongly recommended to provide a comprehensive assessment of vascular involvement [184]. Early identification is a critical step in the management of TIAs, particularly in high-risk patients, such as those in the early postoperative period following aortic arch surgery. Among antiplatelet therapies, clopidogrel alone has been shown to significantly reduce the risk of major vascular events while presenting a lower risk of bleeding in high-risk populations, including patients with prior cardiac surgery [185]. This makes clopidogrel the preferred choice for post-aortic arch surgery patients. In contrast, dual antiplatelet therapy with aspirin and clopidogrel has not demonstrated a significantly greater reduction in major vascular complications but is associated with an increased risk of severe bleeding, including intracranial hemorrhage and other life-threatening events [185].

### Rehabilitation and long-term management

Evidence indicates that while surgical outcomes for aortic arch procedures generally improve over time, many patients continue to report lower physical and mental well-being than the general population, highlighting the importance of consistent postoperative follow-up. Regular monitoring is essential for detecting and managing secondary complications, such as reoperation requirements and aortic aneurysm formation, especially in complex cases involving hybrid or open repair techniques [186]. Comprehensive follow-up requires scheduled imaging, such as CT, MRI, or echocardiography (particularly in cases involving root disease), to monitor the aorta and adjacent structures. Guidelines specify different follow-up protocols based on the underlying condition that led to surgery. For instance, the European Society of Cardiology recommends imaging at 1, 6, and 12 months post-aortic dissection surgery, then annually thereafter, whereas radiographic follow-up for repaired aortic arch aneurysms is suggested at one year, with additional assessments every 2 to 3 years [14]. In addition to imaging, cardiac rehabilitation programs that incorporate physical, cognitive, and psychological support have demonstrated substantial benefits, enhancing endurance, cognitive function, and overall health status. Additionally, integrating rehabilitation programs with cardiac rehabilitation improves physical endurance, cognitive function, and overall health outcomes, addressing long-term neurological or cognitive deficits post-surgery [187].

## Outcomes and prognosis

### Short-term outcomes

Aortic arch surgery carries significant short-term risks, with studies reporting in-hospital mortality rates ranging from 6.52% to 15.9% [73, 147, 159, 188]. One study found that urgent or emergent surgeries had a higher mortality rate (15.1%) compared to elective procedures (9.9%) [73]. Neurological complications are a major concern in the immediate postoperative period. The incidence of PND varied across studies, from 4.2% to 8.7% [73, 147, 159, 189], while TND rates ranged from 12.3% to 40% [73, 147, 159, 189].

The type of cerebral protection technique used during surgery appears to influence outcomes. One study found that TND was significantly more common with retrograde cerebral perfusion compared to antegrade cerebral perfusion (40% vs. 17.39%) [189]. Additionally, the extent of hypothermia during surgery may impact outcomes, with one study suggesting that mild hypothermic circulatory arrest combined with sACP may reduce the incidence of major adverse events compared to moderate hypothermia [159].

SCI is another serious complication, with one study reporting a 7.5% incidence [188]. Several factors were identified as predictors of poor short-term outcomes, including advanced age, female gender, higher EuroSCORE, chronic renal failure, more extensive aortic replacement, longer CPB time, preoperative hemodynamic instability, peripheral vascular disease, diabetes, and antegrade selective cerebral perfusion time exceeding 60 min [73, 188]. Specific surgical techniques, such as arterial cannulation of the ascending aorta and a distal landing zone lower than T10 in FETs, were also associated with increased risk of stroke and SCI, respectively [188].

### Long-term functional status

The long-term functional status of patients who survive aortic arch surgery appears generally favorable, though data is limited and outcomes can vary. In a pediatric population, one study reported that at a mean follow-up of 48.9 months, all surviving children had normal functional status and were free of cardiac symptoms [190]. However, two patients in this cohort had residual neurological sequelae identified during the perioperative period, including controlled seizures, motor/cognitive delays, and speech delay [190].

For adult patients, the picture is more complex. A study comparing patients who underwent more extensive aortic arch surgery (hemiarch or TAR) to those who had only ascending aorta replacement found poorer long-term outcomes in the former group [191]. These patients had a higher rate of new postoperative neurological deficits (17.5% vs. 8.4%), more frequent strokes (15.7% vs. 6.5%), lower QoL scores for both physical and mental components after long-term follow-up, and worse long-term survival [191].

Another study evaluating long-term QoL in patients who underwent replacement of the ascending aorta found a significant decline in both physical and mental component summary scores compared to the normal population [192]. Interestingly, the extent of the surgical procedure (isolated supracoronary replacement vs. more complex procedures involving the aortic root/valve) did not significantly influence postoperative QoL in this study [192]. Patients showed some improvement in several QoL domains during follow-up compared to one year post-surgery, suggesting potential for recovery over time [192].

It’s important to note that while most cases of transient neurological dysfunction resolve completely “after some days or weeks” [189], the long-term impact of these temporary deficits on cognitive function and QoL is not well-established in the available literature. The studies generally suggest that the majority of patients who survive the immediate postoperative period without PND can achieve good functional outcomes. However, the potential for reduced quality of life, particularly in mental health aspects, highlights the need for ongoing psychosocial support and long-term follow-up for these patients [192].

These findings suggest that while many patients achieve good functional outcomes after aortic arch surgery, the extent of the procedure and the occurrence of perioperative neurological complications can significantly impact long-term functional status. Further research with longer follow-up periods and specific assessments of functional status is needed to better understand the long-term prognosis for these patients.

### QoL considerations

Several studies have examined QoL outcomes following aortic arch surgery, with mixed results. One study using the SF-36 questionnaire found that patients showed significant declines in both physical and mental component summary scores compared to the normal population [192]. Interestingly, the extent of the surgical procedure did not significantly influence postoperative QoL in this study [192]. Another study using the Sickness Impact Profile (SIP) questionnaire reported excellent overall QoL scores at a median follow-up of 28 months, with patients reporting completely normal QoL in 8 out of 12 categories [193]. However, patients who suffered a stroke had significantly worse scores in both physical and psychosocial dimensions [193].

The impact of surgical techniques on QoL has also been explored. One study found that for circulatory arrest times up to 20 min, QoL scores were similar across all groups and within the range of an age/sex-matched standard population [194]. However, longer circulatory arrest times were associated with impaired QoL, particularly in patients without cerebral protection [194]. The use of right axillary artery cerebral perfusion appeared to provide the best protection for QoL outcomes, allowing circulatory arrest times to be extended up to at least 50 min without impairing self-reported QoL compared to the general population [194].

It’s important to note that while some studies found comparable overall QoL to age- and sex-matched reference groups [195], others reported lower scores in specific domains such as physical functioning, general health, and mental health [195]. Current symptoms like exertional dyspnea were found to be independently associated with worse QoL scores, rather than operative factors [195]. This suggests that optimizing the management of ongoing symptoms and comorbidities may be crucial for improving the long-term QoL in these patients.

To conclude, while aortic arch surgery can be performed with acceptable short-term mortality and neurological complication rates, there remains a significant risk of adverse outcomes. Long-term functional status is generally favorable for many patients but can be impacted by the extent of surgery and perioperative complications, particularly strokes. QoL outcomes are variable, with some studies reporting good overall QoL and others noting impairments in specific domains. Surgical techniques, particularly those offering enhanced cerebral protection, may play a role in preserving long-term QoL. Ongoing management of symptoms and comorbidities appears to be crucial for optimizing long-term outcomes and QoL for these patients.

## Future directions

The field of aortic arch surgery continues to evolve with the goal of minimizing neurological complications while improving patient outcomes. Recent advances, including hybrid surgical approaches and robotic-assisted techniques, offer promising solutions but warrant further exploration.

### Hybrid surgical approaches

The hybrid approach, combining traditional open surgical repair with minimally invasive endovascular techniques, is emerging as a vital strategy. While open surgical repair remains the gold standard, hybrid repairs (HR) offer reduced invasiveness, making them particularly beneficial for patients with complex aortic anatomy [196, 197]. Although the neurological outcomes, such as stroke and paraplegia, are comparable between open surgical repair and hybrid repair, hybrid approaches tend to demonstrate lower morbidity and mortality, making them a valuable alternative in specific cases [198]​.

### Robotic-assisted surgery

Robotic-assisted procedures are gaining traction in cardiovascular surgery, particularly for high-risk patients. Although robotic-assisted surgery has shown significant benefits in terms of cosmetic outcomes, shorter recovery times, and reduced surgical stress [199–201], its applicability in aortic surgery is still under investigation. Studies suggest that combining robotic assistance with endovascular techniques could enhance safety, improve precision, and reduce operative time [202]. However, challenges like higher costs and longer procedural times limit its widespread adoption [200, 203]. More randomized trials are necessary to confirm the long-term benefits of robotic surgery in this field.

### Cerebral protection: hypothermia and Pharmacological interventions

Accurate brain temperature monitoring during aortic arch surgery is crucial for protecting against neurological complications. While deep hypothermia has long been used to protect the brain by lowering metabolic rates, it is associated with risks such as brain injury. Recent studies indicate that moderate hypothermia combined with antegrade cerebral perfusion may provide equivalent cerebral protection while reducing complications [168, 204]​.

Pharmacological strategies, such as the use of steroids to mitigate cerebral swelling and inflammation, are another area of ongoing research. While steroids are routinely employed, their efficacy in protecting the brain during aortic arch surgery is still debated [205]. Alternative neuroprotective agents targeting cerebral metabolism could offer new avenues for reducing neurological deficits post-surgery.

### Standardization and collaborative research

One significant barrier to advancing research in aortic arch surgery is the lack of standardized protocols and data reporting. Multi-center, randomized controlled trials are essential to eliminate selection bias and provide clearer insights into the most effective surgical and cerebral protection techniques​ [196]. Future studies should focus on creating a consensus around optimal brain protection strategies, incorporating diverse patient populations, and comparing long-term neurological outcomes. Additionally, multi-center RCTs should aim to directly compare moderate hypothermia with sACP versus deep hypothermia in patients undergoing total arch replacement, with standardized neurological and cognitive testing at 1 and 5 years. Such efforts will be critical to determine the true long-term benefits and risks associated with each cerebral protection strategy and to guide evidence-based clinical decision-making.

In conclusion, the future of aortic arch surgery lies in refining hybrid and robotic-assisted techniques, optimizing cerebral protection strategies, and fostering collaborative research. Addressing these challenges will pave the way for improved patient outcomes and reduced neurological complications in the years to come.

## Limitations

Despite comprehensive database searches and manual screening of reference lists, there remains a possibility that some relevant studies were not identified. The decision to include only articles published in English may have introduced language bias and excluded important findings reported in other languages. Furthermore, internal limitations of the review process, including potential selection and interpretive biases, cannot be entirely ruled out.

## Conclusion

Neurological complications following aortic arch surgery remain a significant concern, despite advances in surgical techniques and perioperative care. This review focuses on the most clinically relevant complications, including stroke, TIAs, seizures, spinal cord injury, and postoperative cognitive dysfunction. These adverse outcomes significantly affect patient well-being by increasing morbidity and mortality, prolonging hospital stays, and reducing overall quality of life. The aforementioned complications are multifactorial in nature and are presumed to be due to pathophysiological causes such as cerebral hypoperfusion, embolism, systemic inflammatory response, and reperfusion injury. Successful outcomes rely on a multidisciplinary approach of preoperative risk stratification, intraoperative neuroprotection, and meticulous postoperative surveillance. While established neuroprotective strategies, such as sACP, hypothermia, and advanced monitoring techniques (e.g., NIRS and EEG), have shown promising results, there remains a persistent gap in their ability to ensure consistent neuroprotection and support long-term recovery. This underscores the need for future research and innovative interventions aimed at optimizing surgical techniques and exploring novel pharmacologic agents. A key objective should be the improvement of long-term QoL for patients undergoing these complex, high-risk procedures.

## Data Availability

No new data was generated.

## Abbreviations

PND: Permanent neurological deficits/Dysfunction
TND: Temporary/Transient neurological deficits
DHCA: Deep hypothermic circulatory arrest
CPB: Cardiopulmonary bypass
IMH: Intramural hematoma
LVA: Left vertebral artery
ASA: Anterior spinal artery
TAR: Total arch replacement
ET: Elephant trunk technique
FET: Frozen elephant trunk procedure
TEVAR: Thoracic endovascular aortic repair
NIRS: Near-infrared spectroscopy
TIA: Transient ischemic attack
SCI: Spinal cord injury
CSFD: Cerebrospinal fluid drainage
POCD: Postoperative cognitive dysfunction
sACP: Selective antegrade cerebral perfusion
rScO2: Regional cerebral oxygen saturation
EEG: Electroencephalography
TCD: Transcranial doppler
CSF: Cerebrospinal fluid
QoL: Quality of life
